# The Swedish childhood tumor biobank: systematic collection and molecular characterization of all pediatric CNS and other solid tumors in Sweden

**DOI:** 10.1186/s12967-023-04178-4

**Published:** 2023-05-23

**Authors:** Teresita Díaz de Ståhl, Alia Shamikh, Markus Mayrhofer, Szilvester Juhos, Elisa Basmaci, Gabriela Prochazka, Maxime Garcia, Praveen Raj Somarajan, Katarzyna Zielinska-Chomej, Christopher Illies, Ingrid Øra, Peter Siesjö, Per-Erik Sandström, Jakob Stenman, Magnus Sabel, Bengt Gustavsson, Per Kogner, Susan Pfeifer, Gustaf Ljungman, Johanna Sandgren, Monica Nistér

**Affiliations:** 1 Department of Oncology-Pathology, Karolinska Institutet, Stockholm, Sweden; 2 Clinical Pathology and Cancer Diagnostics, Karolinska University Hospital, Stockholm, Sweden; 3 Department of Cell and Molecular Biology, National Bioinformatics Infrastructure Sweden, Science for Life Laboratory, Uppsala University, Uppsala, Sweden; 4 Department of Paediatric Haematology Oncology and Immunology, Skåne University Hospital Lund, Lund, Sweden; 5 Department of Clinical Sciences Lund, Department of Neurosurgery, Lund University, Skåne University Hospital, Lund, Sweden; 6 Department of Clinical Sciences, Pediatrics, Umeå University, Umeå, Sweden; 7 Childhood Cancer Research Unit, Department of Women’s and Children’s Health, Karolinska Institutet, Stockholm, Sweden; 8 Childhood Cancer Centre, Queen Silvia Children’s Hospital, Sahlgrenska University Hospital, Gothenburg, Sweden; 9 Department of Neurosurgery, Karolinska University Hospital, Stockholm, Sweden; 10 Pediatric Hematology/Oncology, Department of Women’s and Children’s Health, Uppsala University, Uppsala, Sweden

**Keywords:** Biobank, Genomic, Childhood cancer, Next generation sequence, Mutation, Bioinformatics, Methylation

## Abstract

The Swedish Childhood Tumor Biobank (BTB) is a nonprofit national infrastructure for collecting tissue samples and genomic data from pediatric patients diagnosed with central nervous system (CNS) and other solid tumors. The BTB is built on a multidisciplinary network established to provide the scientific community with standardized biospecimens and genomic data, thereby improving knowledge of the biology, treatment and outcome of childhood tumors. As of 2022, over 1100 fresh-frozen tumor samples are available for researchers. We present the workflow of the BTB from sample collection and processing to the generation of genomic data and services offered. To determine the research and clinical utility of the data, we performed bioinformatics analyses on next-generation sequencing (NGS) data obtained from a subset of 82 brain tumors and patient blood-derived DNA combined with methylation profiling to enhance the diagnostic accuracy and identified germline and somatic alterations with potential biological or clinical significance. The BTB procedures for collection, processing, sequencing, and bioinformatics deliver high-quality data. We observed that the findings could impact patient management by confirming or clarifying the diagnosis in 79 of the 82 tumors and detecting known or likely driver mutations in 68 of 79 patients. In addition to revealing known mutations in a broad spectrum of genes implicated in pediatric cancer, we discovered numerous alterations that may represent novel driver events and specific tumor entities. In summary, these examples reveal the power of NGS to identify a wide number of actionable gene alterations. Making the power of NGS available in healthcare is a challenging task requiring the integration of the work of clinical specialists and cancer biologists; this approach requires a dedicated infrastructure, as exemplified here by the BTB.

**Supplementary Information:**

The online version contains supplementary material available at 10.1186/s12967-023-04178-4.

## Introduction

The importance of sample collections and patient-associated data in the current era of data-driven medical research is substantial, as these data provide a fundamental scientific basis for emerging multifaceted approaches required for personalized treatments. The Swedish Childhood Tumor Biobank (BTB) was established in Sweden to increase our knowledge of the molecular, cellular and genetic diversity of pediatric central nervous system (CNS) and other non-CNS solid tumors. The BTB is an infrastructure based on a national multidisciplinary network that handles the collection and biobanking of biological specimen and the generation of deep genomic data. The samples will be shared for use in research projects after medicolegal review and approval via a Scientific Prioritization Committee. Controlled access to genomic datasets is determined by a Data Access Committee.

Pediatric cancer includes a heterogeneous set of diseases that can be largely divided into leukemias and lymphomas, CNS tumors, and other non-CNS solid tumors [47]. Childhood tumors are challenging to study and treat because they are relatively rare and originate in developing organs in which cytotoxic therapies can have detrimental effects. In Sweden, approximately 350 children up to the age of 18 are diagnosed with cancer each year. Approximately one-third of these children are diagnosed with leukemia and lymphoma, one-third with CNS tumors and another third with various non-CNS solid tumors (https://www.cancercentrum.se/samverkan/cancerdiagnoser/barn/). Treatment advances have raised cure rates for pediatric cancer in high-income countries to approximately 80%; however, survivors often experience major lifelong developmental and cognitive sequelae, including a risk for second malignancies. Moreover, cancer remains a major cause of disease-related death among children [46].

During the last decade, the application of massively parallel sequencing or next-generation sequencing (NGS) has enormously increased our understanding of the molecular mechanisms driving pediatric cancer and contributed to the discovery of novel clinically relevant cancer subtypes. Numerous recent studies highlight that the biology and genetics of pediatric cancer differ from the equivalent adult cancer forms and underscore that the number of somatic mutations in most pediatric cancers is considerably lower than that in adults, with the vast majority of childhood tumors lacking a high mutational burden [27, 47]. However, additional studies have elucidated that childhood cancer genomes are characterized by remarkable heterogeneity in the types of genetic alterations that likely drive tumor growth, including copy number aberrations, structural alterations, chromoplexy and chromothripsis; these modifications can be used as prognostic markers in specific cancer subtypes [9, 15, 30, 36]. Many of these alterations lead to gene fusion events, and new technologies reveal a continuously increasing number of fusion partners that function as oncogenic drivers in many pediatric cancers [15, 30, 36]. Additionally, germline mutations in a wide spectrum of genes appear to play a larger role in childhood cancer predisposition than previously appreciated [39, 53]. Therefore, appropriate and sensitive approaches for identifying a wide and complex spectrum of alterations at both the germline and somatic levels are needed for the meaningful molecular characterization of pediatric cancers.

Here, we describe the complete workflow of the BTB, from the collection and processing of biospecimens to the generation of omics data. We have also analyzed whole genome (WGS) and whole exome sequencing (WES) data obtained retrospectively from a set of samples referred to here as the “pilot subset”, comprising 82 biobanked CNS tumors and patient-matched peripheral blood-derived DNA from 79 children and identified germline and somatic alterations with potential biological or clinical significance to demonstrate the potential utility of the findings in patient management.

## Materials and methods

### I*nformed consent process, BTB samples and data collection*

**Fig. 1:**
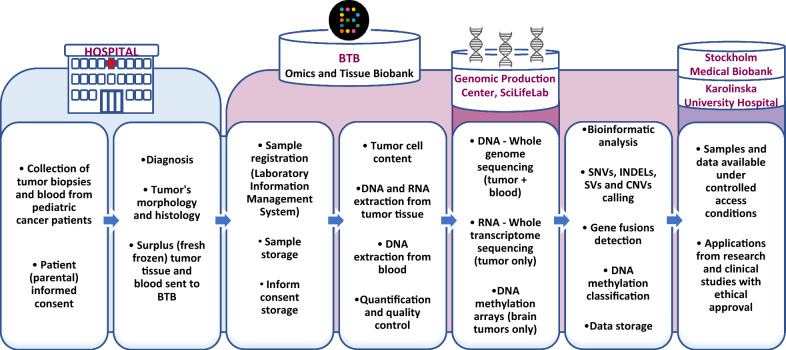
Overview of the BTB sample workflow, from tissue collection to sample and data sharing

Biospecimens from pediatric patients diagnosed with CNS and other solid tumors in Sweden are systematically collected, controlled by pathologist, processed and in-depth genomic data is generated. Both samples and data are stored for long-term at the BTB. An overview of the sample workflow hierarchy is depicted in Fig. 1. Further description on the established procedures is available in Additional file 1: data 1.

### Sequencing and methylation profiling of a pilot subset of BTB samples

CNS tumors are clinically and biologically highly diverse, and this complexity is reflected by the high number of entities defined in the World Health Organization (WHO) classification of CNS tumors [29]. The pilot subset of samples investigated here included 82 fresh frozen tumor samples and patient-matched blood-derived DNA collected from 2014 to 2016 from 79 patients with brain tumors. Samples were selected according to the availability of material and to represent all major pediatric brain tumor types, Additional file 2: Table S1. The data processed included WGS from 137 samples (70 tumors and 67 blood samples) and WES from 24 samples (12 tumors and 12 blood samples). Libraries for WGS were prepared with Illumina TruSeq PCR-free (126 samples, runs P2233, P4551 and P7708). The Illumina TruSeq Nano, NeoPrep protocol was used for 11 samples (run P4552) from which a lower DNA amount was obtained. WGS samples were sequenced 2 × 150 bp paired-end, on a HiSeqX v2.5 instrument, RRID:SCR_016385 (Illumina, San Diego, California). Libraries for WES (11 tumors and matched blood, runs P1148P1 and P1315) were prepared using the Agilent SureSelect Human AllExonV5 protocol (Agilent). Additionally, two samples (FAM2 matched tumor and blood) were exome sequenced using the Twist Human Core Exome Kit (Twist) (https://www.twistbioscience.com/products/ngs#product-featured-1951). WES samples were sequenced 2 × 100 bp paired-end, on a HiSeq 2500 instrument (Illumina). The preparation of libraries and sequencing were performed at the Genomic Production Center, SciLifeLab, Stockholm, Sweden. Sequence data were delivered in FASTQ format using Illumina 1.8 quality scores. The metrics for this pilot subset are summarized in Additional file 3: Table S2. Tumor DNA methylation profiling was performed at the SNP&SEQ Technology Platform, Uppsala, Sweden (www.genotyping.se), using Illumina Infinium Human Methylation 450 or EPIC Bead Chip arrays (Illumina). Five hundred nanograms of DNA derived from fresh-frozen tumors was used as input material. The classifier developed by The German Cancer Research Center (DKFZ classifier, molecularneuropathology.org), a powerful tool to assist in the assignment of CNS tumors into distinct groups/subgroups [5], was applied to the DNA methylation data. IDAT files were processed at DKFZ and assigned to methylation classes using the brain tumor methylation classifier v11b2, Additional file 2: Table S1.

### Sequence data analysis

DNA sequence data were processed via Sarek, an open-source workflow for short read alignment and processing, following the Genome Analysis Toolkit (GATK) best-practice recommendations [13], on UPPMAX Clusters at Uppsala University (https://www.uppmax.uu.se/resources/systems/the-bianca-cluster/). Briefly, the steps were as follows: quality control of FASTQ files using FASTQC (https://www.bioinformatics.babraham.ac.uk/projects/fastqc/), alignment of short reads to the human reference genome sequence (GRCh38/hg38) using bwa-mem, RRID:SCR_022192, with the ALT-aware option turned on (https://arxiv.org/abs/1303.3997), sorting of reads and marking of PCR duplicates with GATK (RRID:SCR_001876). MarkDuplicates, base quality score recalibration and joint realignment of reads around insertions and deletions (indels) were conducted using GATK tools (https://github.com/broadinstitute). Variant calling and annotation, including germline and somatic small-scale variations (SNVs and indels), structural variations (SVs), fusion genes and copy number variations (CNVs), were determined using the Sarek pipeline [13]. HaplotypeCaller from GATK (https://www.biorxiv.org/content/10.1101/201178v3) was run on both the normal and tumor samples, and SNVs and indel calls were generated by Strelka2, RRID:SCR_005109 [23] and GATK MuTect2, RRID:SCR_000559 (https://www.biorxiv.org/content/10.1101/861054v1).

Both germline and somatic SVs were produced by Manta [7]. Only variants marked as “PASS” were considered. Variant annotation was performed with snpEff, RRID:SCR_005191 [8] and VEP [33]. Furthermore, copy number profiles, ploidy and calculated tumor cell ratios were generated by ASCAT, RRID:SCR_016868 [50] and Control-FREEC, RRID:SCR_010822 [3]. We focused on detecting mutations in genes reported to be involved in cancer, including cancer census genes (Cosmic v90) and genes commonly altered in pediatric tumors identified in previous studies [15, 30, 31, 36]. Quality control metrics and coverage were collated by MultiQC, RRID:SCR_014982 [11]. Software versions used are shown in Additional file 4: Table S3. Read alignments were inspected using the Integrative Genomics Viewer, RRID:SCR_011793 [42].

### Samples and data availability

Samples and genomic data (FASTQ, BAM, IDAT, and VCF files) can be requested by approved projects conducting research in the field of pediatric cancer. Applications from researchers are evaluated by the Scientific Priority Committee, which considers the quality and feasibility of the projects and the best use of resources (including tissue and/or DNA/RNA vs. data availability) and recommends the approval (or refusal) of the submissions. Access to biospecimens is contracted by the legally responsible biobank organization, Stockholm Medical Biobank (SMB), Stockholm Region (https://www.eithealth-scandinavia.eu/biobanks/stockholm-medical-biobank-smb/). Controlled access to genomic data and personal information is granted by Karolinska University Hospital (via Data Access Committee).

## Results

### The Swedish childhood tumor biobank (BarnTumörBanken; BTB): a combined genomics and tissue biobank for pediatric cancers

The BTB is a translational research core facility for sample and genomic data collection from pediatric patients diagnosed with solid tumors in Sweden. The BTB is financed by The Swedish Childhood Cancer Fund (BCF) (https://www.barncancerfonden.se/en/) and is located at The Karolinska University Hospital and Karolinska Institutet in Stockholm. The BTB is built on a multidisciplinary nationwide network of physicians and researchers from the six university hospitals that manage the care of affected children. The BTB operates under standard operating procedures to ensure that biospecimens are collected and processed consistently and with high quality. It also maintains strict ethical standards and adheres to national and international regulations for biobanking. The daily work, from sample collection to data generation and storage, is conducted by qualified personnel with relevant expertise. Competence in different working areas to support the administrative and legal procedures also exists. The infrastructure is governed by a steering group that oversees BTB’s activities and takes strategic decisions, including the main goals and research services offered., The members of the steering group represent multidisciplinary clinical and research specialties in the field of pediatric cancer, the patient organization and the BCF.

#### BTB services

The BTB offers services to childhood cancer research projects and clinical studies in compliance with current ethical and legal regulations. Samples and genomic data can be requested by approved projects undertaking meaningful research in the field of pediatric cancer.

Offering services to clinical studies, the BTB has been responsible for the handling of clinical samples for Swedish patients referred to the INFORM (INdividualized Therapy FOr Relapsed Malignancies in Childhood, Germany), CMS (Cerebellar Mutism Syndrome, Denmark), BIOMEDE (Biological Medicine for Diffuse Intrinsic Pontine Glioma Eradication, France), SIOP PNET5 MB (ClinicalTrials.gov Identifier: NCT02066220) and Genomic Medicine Sweden (GMS, Childhood cancer|Genomic Medicine Sweden) studies in recent years [14, 52]. The BTB´s personnel conduct sample collection, DNA and RNA extraction, sample logistics and quality control. As of March 2022, 217 cases have been submitted to INFORM, 155 to CMS, 15 to BIOMEDE, 19 to PNET5 and 145 to GMS. Customized collection or specialized processing of tissue and molecular data for prospective studies or clinical trials is also supported upon formal agreement. The BTB requests funds only to cover personnel time and consumables for the generation of research-ready specimens and datasets.

#### BTB sample and data collection is continuously expanding

**Fig. 2:**
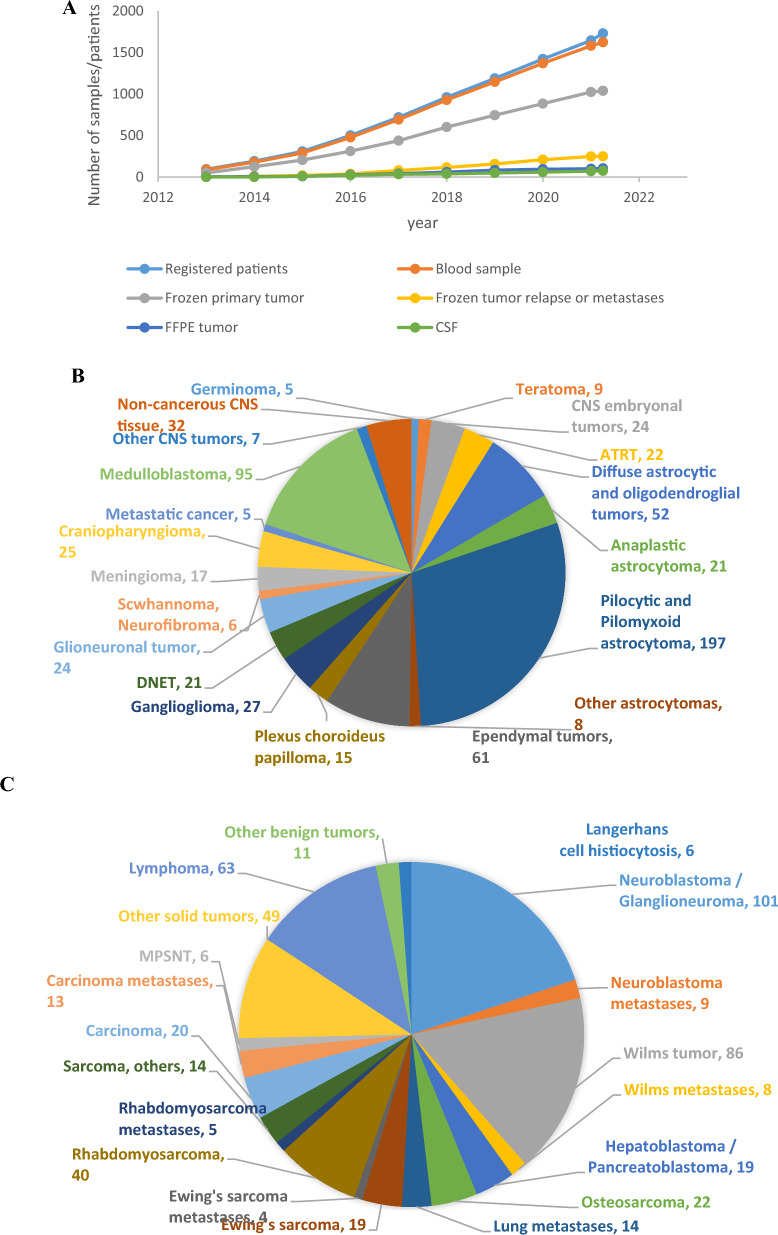
**A** Sample collection at the BTB specifying the cumulative numbers of patients registered at the BTB from whom at least one biological sample (blood, fresh frozen or FFPE tissue) has been collected, fresh frozen primary tumors, patient blood samples, fresh frozen tumor relapses or metastasis and FFPE tumors. Distribution of diagnosis of CNS (**B**) and other solid (**C**) tumors from which samples of fresh frozen primary, relapsed or metastatic tissue and patient-matched blood were collected until March 2022

Biospecimens from affected children are continuously collected for the BTB on a national level. Tissue sampling from CNS and other non-CNS solid tumor patients was initiated in 2013 and 2015, respectively, and since 2020, specimens from approximately 90% of all diagnosed pediatric cancer cases in Sweden have been provided to the BTB. Currently, there are 597 CNS and 407 other non-CNS solid fresh frozen primary tumors and 183 samples with known or suspected tumor relapse in the BTB repository, all sampled with patient-matched blood. Occasionally, additional samples, including Formalin-Fixed Paraffin-Embedded (FFPE) tissue, needle aspirates or cerebrospinal fluid, are obtained. Blood samples from the parents of affected children are also being collected. As of 2022, there are over 1700 registered patients from whom at least one biological sample has been collected (Fig. 2). Large-scale genomic data is generated from the samples. Currently, WGS and transcriptome sequencing are retrospectively performed on frozen tumors, complemented with DNA methylation profiling for CNS tumors to enhance diagnostic accuracy. WGS is also performed on patient-matched blood-derived DNA.

### The genomic landscape of pediatric brain tumors: analysis of somatic and germline mutations in a pilot subset of BTB samples

**Fig. 3:**
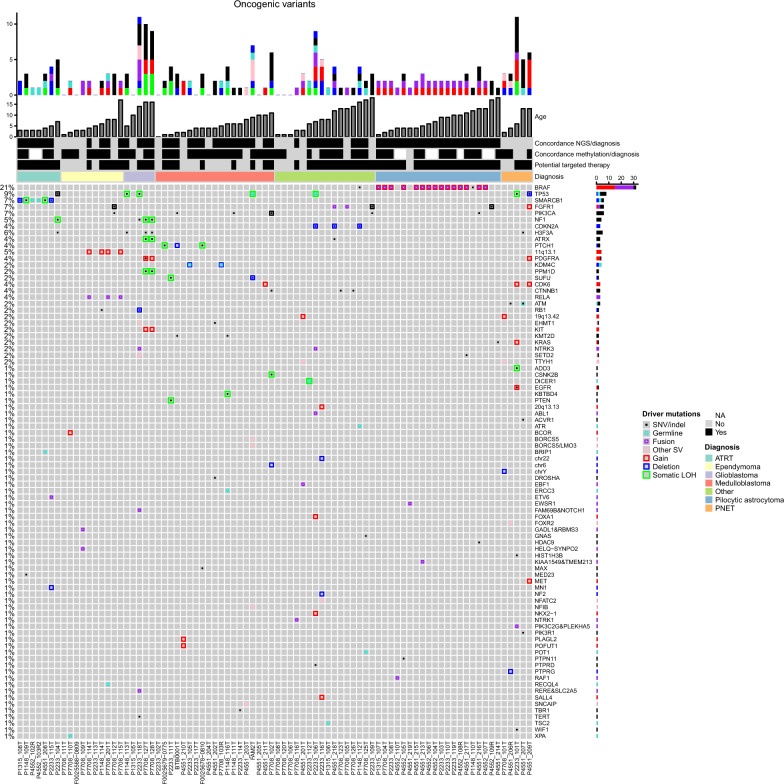
Oncoprint of genomic alterations in CNS tumors. Heatmap of genomic alterations in the series of 82 tumors. Individual genes are represented as rows, and tumors are represented as columns. The bars at the top of the figure indicate the number of cumulative events (putative drivers) identified in the corresponding sample at baseline. The colors represent the six types of underlying genomic alterations as indicated on the right. Concordance between methylation classification or NGS results and pathology diagnosis, the potential availability of targeted therapies, and the pathology diagnosis (indicated in different colors) are also shown. The genes (rows) are sorted by their alteration frequency in the cohort, as noted on the left. When two events affect the same gene in a sample, they are represented in the same slot. Different colors and sizes of rectangles distinguish the type of alterations, as indicated in the panel on the right

It is fundamental that biospecimens and data collected at the BTB can successfully support basic and translational biomedical research. To evaluate the results of the whole workflow and demonstrate the collection’s value, we outline here the overall findings derived from basic bioinformatic analyses performed on omics data from a series of pediatric brain tumor samples collected and sequenced during the first stage of the project. We analyzed data generated from WGS or WES on a total of 82 brain tumors and peripheral blood samples from 79 affected children (Additional file 2: Table S1, Fig. 3, Additional file 5: Fig. S1, Additional file 6: Fig. S2). These included 19 diagnosed medulloblastomas (MB), ten ependymomas, 11 glioblastomas (GBMs)/primitive neuroectodermal tumors (PNET)/embryonal tumors, 20 pilocytic astrocytomas (PAs), five representing other forms of astrocytoma/glioma, seven atypical teratoid rhabdoid tumors (ATRT), two oligodendrogliomas, two meningiomas, two craniopharyngiomas, and one each of pineoblastoma, ganglioglioma, pituitary adenoma, choroid plexus tumor, and schwannoma. Patient age ranged from one month to 18 years (median 5.8 years).

The DKFZ classifier was applied to the DNA methylation data. The methylation-based classification agreed with or improved the pathologic diagnosis by confidently assigning the tumors into specific classes in 78% of analyzed cases (57 of 73 profiled tumors with classification scores > 0.9; Additional file 2: Table S1). Five additional tumors were assigned to methylation class families with lower scores (values ranging from 0.49–0.74), and in seven cases, the methylation screening failed to classify the samples or scored poorly. Some of these cases represent tumors with lower tumor cell content, while others may represent rare or uncharacterized entities or subgroups that are likely underrepresented in the reference cohort used to train the classification model.

Moreover, while accounting for molecular subgroups is important in itself, to enable the development of more appropriate therapeutic strategies and meaningful clinical trials, it is essential to determine the underlying mutation pattern of disease-relevant genes. Therefore, WGS or WES was performed on the samples. Relevant germline and somatic mutations and methylation classification results are described below and summarized in Additional file 2: Table S1.

#### Previously recognized genomic alterations in different diagnostic entities

Medulloblastoma (MB) is the most common malignant pediatric brain tumor in children. It represents a biologically and clinically heterogeneous disease comprising several molecularly distinct subgroups with clinical and genetic differences. Since 2012, the general consensus has been that MB can be molecularly separated into at least four core subtypes, including WNT-activated, sonic hedgehog (SHH)-activated, MB group 3 and group 4 [49]. The methylation profiling of MB confidently determined the subclasses in 16 cases and in one additional case but with a lower score (P4551_203T). This included one WNT, five group 3, six group 4 and five SHH tumors. Mutations in subgroup-restricted genes were found; for example, one MB-WNT (P7708_102T) tumor presented with the typical *CTNNB1* mutation, three (F0025678-0810, F0025679-0775, BTB0001) SHH-MB were driven by mutation/deletion of *PTCH1*, and two (FAM2T, P2233_111T) were driven by *SUFU* mutation/deletion in combination with *TP53* or *PTEN* mutation. Additionally, one MB group 4 tumor (P2233_114T) presented a *TBR1* mutation, another (P4551_203T) carried a tandem duplication of the *SNCAIP* gene, which leads to *PRDM6* activation and is the most common distinctive genomic alteration described for MB group 4 [37], and one MB group 3 tumor (P1148_116T) harbored the hotspot insertion in *KBTBD4* [36]. Mutations in genes known to be mutated across the subgroups, including *PIK3CA* and *KMT2D,* were also detected (Additional file 2: Table S1).

Pediatric high-grade gliomas (HGGs), including diffuse intrinsic pontine glioma (DIPG) and GBM, are among the most aggressive brain tumors. IDH wild-type HGG comprises eight molecular subtypes determined by DNA methylation profiling (K27, G34, RTK I, RTK II, RTK III, mesenchymal, midline, MYCN) that are strongly associated with clinical features (https://www.molecularneuropathology.org/mnp). Among the samples studied here, four tumors from three patients were classified as diffuse midline glioma K27 mutant (P2233_104T, P1148_113T, P7708_127T, P7708_128T) and harbored mutations in *H3F3A* in addition to *TP53* or *ATRX* mutations. The fifth GBM, classified as IDH wild-type, subclass MYCN tumor (P4551_209T), carried *TP53* mutations and several amplified loci, including *FGFR1*, *CDK6*, *MET* and *PDGFRA,* but not *MYCN* (Fig. 3, Additional file 2: Table S1, Additional file 6: Fig. S2).

Ependymal tumors are neuroepithelial malignancies of the CNS that occur in both children and adults. Despite the histopathological similarities among variants of ependymomas at different anatomical sites, the molecular biology of this tumor is heterogeneous, and nine molecular subgroups with distinct genetic and epigenetic alterations have been identified [38]. In accordance with this knowledge, no drivers were found in the three ependymoma posterior fossa group A tumors studied here (F0025588-0809, P2233_113T, P7708_111T), and all four ependymomas in the RELA fusion class (P1148_114T, P7708_114T, P7708_115T, P7708_201T) displayed copy number alterations at 11q13.1 involving C11orf95 and RELA (Table 1, Additional file 2: Table S1, Additional file 5: Fig. S1, Additional file 6: Fig. S2G).

**Table 1:** Somatic structural variants predicting fusion genes

Sample	Consequence	Type of SV^1^	Transcript1::Transcript2^2^	Start-Stop (GRCh38 coordinates)	Pathological diagnosis
P2233_115T	*ETV6::NTRK3*	BND	*ENST00000396373.8|5/7::ENST00000626019.2|12/18*	chr12:11880139-chr15:88000019	ATRT
P7708_114T	*C11orf95::RELA*	DUP	*ENST00000433688.2|3/5::ENST00000406246.7|2/11*	chr11:63764600-chr11:65662555	Ependymoma
P7708_115T	*C11orf95::RELA*	double BND	*ENST00000433688.2|3/5::ENST00000406246.7|2/11*	chr11:63764612-chr11:65662560	Ependymoma
P7708_201T	*C11orf95::RELA*	DUP	*ENST00000433688.2|2/5::ENST00000406246.7|2/11*	chr11:63765733-chr11:65663414	Ependymoma
P7708_109T	*GADL1::RBMS3*	INV	*ENST00000282538.9|12/15::ENST00000636680.1|6/17*	chr3:29516136-chr3:30793136	Ependymoma
	*SYNPO2::HELQ*	INV	*ENST00000307142.8|1/5::ENST00000295488.7|9/18*	chr4:83435026-chr4:118898900	
P7708_116T	*ARHGEF2::NTRK1*	INV + DEL	*ENST00000673475.1|25/26::ENST00000524377.6|12/17*	chr1:155949192-chr1:156875269	Ganglioglioma
P2233_118T	*RERE::SLC2A5*	DUP	*ENST00000337907.7|2/24::ENST00000377424.9|12/12*	chr1:8739338-chr1:9087076	
P2233_119T	*KIAA1549::BRAF*	DUP	*ENST00000422774.2|16/20::ENST00000288602.11|9/19*	chr7:138852276-chr7:140790556	Pilocytic astrocytoma
P4551_213T	*KIAA1549::BRAF*	double INV DUP	*ENST00000422774.2|16/20::ENST00000644969.2|9/20*	chr7:138852518-chr7:140791970	Pilocytic astrocytoma
P2233_108T	*KIAA1549::BRAF*	DUP	*ENST00000422774.2|16/20::ENST00000644969.2|9/20*	chr7:138852755-chr7:140789812	Pilocytic astrocytoma
P2233_110T	*KIAA1549::BRAF*	DUP	*ENST00000422774.2|16/20::ENST00000644969.2|9/20*	chr7:138853500-chr7:140792494	Pilocytic astrocytoma
P4552_104T	*KIAA1549::BRAF*	DUP	*ENST00000422774.2|16/20::ENST00000644969.2|9/20*	chr7:138854568-chr7:140790408	Pilocytic astrocytoma
P2233_103T	*KIAA1549::BRAF*	DUP	*ENST00000422774.2|16/20::ENST00000644969.2|9/20*	chr7:138856929-chr7:140789598	Pilocytic astrocytoma
P7708_104T	*KIAA1549::BRAF*	DUP	*ENST00000422774.2|16/20::ENST00000644969.2|9/20*	chr7:138858793-chr7:140791924	Pilocytic astrocytoma
P4552_106T	*KIAA1549::BRAF*	DUP	*ENST00000422774.2|16/20::ENST00000644969.2|9/20*	chr7:138859026-chr7:140791514	Pilocytic astrocytoma
P4552_105T	*KIAA1549::BRAF*	DUP	*ENST00000422774.2|16/20::ENST00000644969.2|9/20*	chr7:138860733-chr7:140788171	Pilocytic astrocytoma
P4551_217T	*KIAA1549::BRAF*	DUP	*ENST00000422774.2|15/20::ENST00000644969.2|9/20*	chr7:138862714-chr7:140787922	Pilocytic astrocytoma
P4552_108R	*KIAA1549::BRAF*	DUP	*ENST00000422774.2|15/20::ENST00000644969.2|9/20*	chr7:138863829-chr7:140793843	Pilocytic astrocytoma
P4551_215T	*KIAA1549::BRAF*	DUP	*ENST00000422774.2|15/20::ENST00000644969.2|9/20*	chr7:138867796-chr7:140792603	Pilocytic astrocytoma
P4551_216T	*KIAA1549::BRAF*	DUP	*ENST00000422774.2|12/20::ENST00000644969.2|9/20*	chr7:138878216-chr7:140787971	Pilocytic astrocytoma
P4551_219T	*EWSR1::PATZ1*	INV	*ENST00000414183.6|9/18::ENST00000266269.10|1/5*	chr22:29289872-chr22:31344672	Pilocytic astrocytoma
P4552_110T	*QKI::RAF1*	BND	*ENST00000361752.8|3/18::ENST00000442415.7|8/18*	chr6:163496728-chr3:12604073	Pilocytic astrocytoma
P7708_105T	*FGFR1::TACC1*	INV	*ENST00000425967.7|18/19::ENST00000317827.9|7/13*	chr8:38413736-chr8:38835597	Oligodendroglioma
P4551_218T	*FGFR1::TACC1*	INV	*ENST00000425967.7|18/19::ENST00000317827.9|7/13*	chr8:38413197-chr8:38833204	Pilocytic astrocytoma anaplastic
P2233_101T	*PLEKHA5::PIK3C2G*	DUP	*ENST00000429027.6|3/32::ENST00000676171.1|19/33*	chr12:18476311-chr12:19180500	PNET
P2233_106T	*ABL1::FLJ37453*	BND	*ENST00000372348.7|1/11::ENST00000317122.2|2/2*	chr9:130847932-chr1:15838837	Rhabdoid meningioma
	*GABRA5::NTRK3*	INV	*ENST00000400081.7|3/11::ENST00000626019.2|9/19*	chr15:26871512-chr15:88134456	
	*MOCOS::ELP2*	DUP	*ENST00000261326.6|6/15::ENST00000442325.6|21/23*	chr18:36168162-chr18:36205353	

^1^Type of Structural Variant: *BND*   breakend notation, translocation, *DUP*  duplication, *INV* inversion

^2^5' and 3' genes and transcripts involved in the fusion transcript followed by the exon involved in the breakpoint and total number of exons in the transcript

ATRTs are highly aggressive embryonic tumors primarily encountered in children with mutations in *SMARCB1* as the main genetic hallmark. We studied seven tumors from six ATRT patients. Methylation profiling provided additional information concerning the subclass for five of these tumors, two being subclass MYC and two subclass TYR. In accordance with previous reports [48], the ATRT-MYC subgroup tumors presented with focal *SMARCB1* deletions (Additional file 2: Table S1, Additional file 5: Fig. S1, Additional file 6: Fig. S2). Furthermore, in one of these tumors (P2233_115T), an *ETV6::NTRK3* fusion was detected (Table 1). The two ATR subgroup TYR presented with a somatic deletion of the whole chr22 and an identical, not previously reported, frameshift SMARCB1:p.Pro392fs mutation. In two relapsed ATRTs (P4552_102R, P4552_103R2) resected on different occasions from the same patient, no mutations were identified apart from a focal deletion within *SMARCB1*, an exon loss variant of germline origin (chr22:23798791–23806662). Pathological re-examination of the slides confirmed low tumor cell content (< 30%) in both cases, explaining why additional genetic abnormalities were not detected. Furthermore, one additional tumor (P2233_104T) pathologically diagnosed as ATRT was unexpectedly assigned by methylation profiling to the diffuse midline glioma, H3 K27M mutant class. NGS findings, including the detection of somatic mutations in the *TP53, NF1* and *H3F3A* genes, strongly supported the methylation classification in this case (Additional file 2: Table S1, Additional file 5: Fig. S1).

Craniopharyngiomas are rare, benign brain tumors of the teratoma type. There are two different subtypes that differ clinically and pathologically: adamantinomatous and papillary craniopharyngioma. These subtypes harbor mutually exclusive and clonal mutations in *CTNNB1* and *BRAF*, respectively [4, 18]. In line with this knowledge, both adamantinomatous craniopharyngiomas profiled here (P7708_123T, P7708_126T) displayed classical mutations in exon 3 of *CTNNB1* (Additional file 2: Table S1).

PA is the most common pediatric brain tumor and is classified as a WHO grade I neoplasm [29]. The most common alteration in PAs is a tandem duplication at 7q34 that results in *KIAA1459::BRAF* fusion [21, 40]. The 7q34 tandem duplication involving the *KIAA1459* and *BRAF* genes was the only CNV observed in 15 of the 20 PAs studied here. The exact breakpoints of these rearrangements were identified as SVs by Manta in 13 of the tumors; of these rearrangements, 12 were tandem duplications, and one (P4551_213T) was a double inversion combined with a duplication. Other mutations were rare, and among them, we detected a pathogenic somatic missense mutation in *PTPN11* in one sample (P4552_105T). Three additional PAs presented with either *KRAS, FGFR1* or other *BRAF* mutations (P4551_214T, P4552_109R, and P1148_110T, respectively), and one carried a rare *QKI::RAF1* fusion (P4552_110T) [20], all leading to MAPK pathway activation.

#### Unique genomic findings previously reported in rare entities

One histopathologically diagnosed PA could not be assigned to a methylation class (P4551_219T). The tumor harbored an *EWSR1::PATZ1* fusion, a rare alteration that appears to define a new type of glioneuronal tumor [43] (Table 1, Additional file 2: Table 1, Additional file 5: Fig. S1, Additional file 6: Fig. S2).

Fusions involving *NTRK* genes are clinically relevant alterations, leading to constitutionally active chimeric receptors with oncogenic potential [1]. We detected chromosomal rearrangements involving *NTRK* genes in ATRT, ganglioglioma and rhabdoid meningioma (one of each; samples P2233_115T, P7708_116T, and P2233_106T; Table 1, Additional file 2: Table S1). Furthermore, the cooccurrence of *FGFR1* and *PIK3CA* hotspot mutations was observed in two tumors pathologically diagnosed as oligodendroglioma and ependymoma (P2233_109T, P7708_112T), and both were assigned to the methylation class low-grade glioma, rosette-forming glioneuronal tumors. These findings align with previous observations indicating that recurrent and combined genetic alterations affecting both MAPK and PI3K signaling pathways appear to interact synergistically during the development of these rare neoplasms [44]. Other interesting alterations involving *FGFR1* detected in this series of tumors were two *FGFR1::TACC1* fusions found in one anaplastic PA (P4551_218T) and one oligodendroglioma (P7708_105T). While the methylation profiling agreed with the histopathological diagnosis in the first sample, no match was found for the second sample (Table 1, Additional file 2: Table S1).

Additional findings derived from the sequencing data that also align with the molecular alterations described within the specific tumor methylation classes included an internal BCOR gene tandem duplication, identified in a CNS high-grade neuroepithelial tumor with a *BCOR* alteration (HGNET-BCOR), diagnosed as ependymoma (P7708_110T); SVs in the vicinity of *FOXR2* in a tumor assigned to the methylation class CNS neuroblastoma with *FOXR2* activation, diagnosed as PNET (P4551_206R); and focal amplifications of the C19MC locus at 19q13.42, involving *TTYH1* in two embryonal tumors with multilayered rosettes (ETMR), diagnosed as PNET and ependymoblastoma, respectively (P7708_101T, P4551_201T; Additional file 2: Table S1, Additional file 5: Fig. S1, Additional file 6: Fig. S2). It is noteworthy to mention that the updated 2016 WHO classification of CNS tumors no longer recognizes PNET as a distinct entity. Molecular analysis has revealed that many tumors previously reported as PNET are now reclassified as defined CNS tumor entities with specific genetic characteristics.

#### Previously unreported alterations observed here

In addition to revealing somatic mutations in a broad spectrum of genes previously implicated in pediatric cancers, we discovered numerous previously unreported events, such as focal amplifications, including *BORCS5* and *LMO3* in an SHH-MB (FAM2T), an amplicon containing the *PLAGL2* and *POFUT1* genes in a sample diagnosed as MB (P4551_210T), *GADL1::RBMS3* and *SYNPO2*::*HELQ* fusions in an ependymoma (P7708_109T) and a *PLEKHA5::PIK3C2G* fusion in a PNET (P2233_101T). These alterations might represent novel driver events (Table 1, Additional file 5: Fig. S1, Additional file 7: Fig. S3). Notably, two of these three tumors could not be classified by methylation arrays, and the third only with a low score, Additional file 2: Table S1.

#### Germline mutations

There is already a clinical demand for germline sequencing data for specific genes. *TP53* status, for example, is of particular relevance, and patients carrying *TP53* germline mutations are at high risk for developing secondary neoplasia following irradiation or DNA-damaging chemotherapy. *TP53* germline mutations were observed in two patients, a splice donor variant in an SHH-MB (FAM2N) and a missense variant in a pathologically diagnosed rhabdoid meningioma (P2233_125N, with no matching methylation class). In both tumors, loss of the wild-type *TP53* allele was observed (Additional file 2: Table S1).

Other relevant germline mutations included a *SMARCB1* deletion in a patient with ATRT (P4551_227N, already discussed above), a novel stop gain mutation in *TSC2* in a patient affected by subependymal giant cell astrocytoma (P1315_102N), a novel frameshift mutation in *KDM4C* accompanied by a somatic exon loss mutation in a child with MB group 3 (P2233_124N), and a *DICER1* nonsense p.Arg509Ter, rs886037672 mutation in a patient with pineoblastoma (P2233_131N). The latter mutation is reported as pathogenic in ClinVar and known to predispose patients to several cancers. Somatic LOH was also observed in the tumor, indicating that *DICER1* acts as a tumor suppressor gene in pineoblastoma (Additional file 2: Table S1).

## Discussion

### NGS of fresh frozen biobanked solid tumor tissue provides a comprehensive interrogation of clinically actionable genomic aberrations

The BTB is a non-profit organization for sample and genomic data collection from pediatric patients diagnosed with solid tumors in Sweden, funded with the central aim of supporting the generation of new scientific knowledge in the field. Unlike traditional tissue repositories, the BTB not only collects biomaterials but also conducts large-scale genomic analysis on the samples. As a result, the BTB represents a state-of-the-art infrastructure for advanced research in pediatric oncology.

The ability of NGS to comprehensively reveal the heterogeneous landscape of genetic alterations in cancer supports its important clinical role as a tool for identifying driver mutations associated with therapeutic targets. To evaluate the results of the whole workflow and demonstrate the collection’s value, analysis of somatic and germline mutations in a pilot subset of BTB samples was performed. Most of the tumors investigated carried aberrations that could be potentially targeted; however, as most therapeutic strategies are not developed for children, severe effects may be observed; for example, growth arrest. Results from pediatric clinical trials are pending in most cases and are required before specific treatments can be implemented. *PIK3CA*-activating mutations and *PTEN* loss-of-function mutations can be targeted by PI3K/AKT/mTOR inhibitors, *CDK6* amplification by CDK4/6 inhibitors and *PTCH1* inactivating mutations in MB by Sonic Hedgehog pathway inhibitors, such as SMO or GLI inhibitors [6, 34]. Histone mutations are highly prevalent in pediatric HGG and confer a poorer prognosis than histone wild-type status. Several drugs, including HDAC inhibitors and the selective dopamine receptor D2 antagonist ONC201, are being investigated in clinical trials for K27M-mutated pediatric glioma (NCT02717455, NCT03416530) [34]. Furthermore, tyrosine kinase inhibitors may be effective for treating pediatric patients with *PDGFRA* mutations or alterations in other tyrosine-kinase receptors, including *FGFR* [34]. Detection of chromosomal rearrangements involving *NTRK* genes is clinically highly relevant, as these rearrangements lead to constitutionally active chimeric receptors with oncogenic potential [1]. Importantly, therapeutic targeting of *NTRK* activation has recently shown substantial efficacy, resulting in significant tumor shrinkage and prolonged progression-free survival [12]. As a result, it has become a promising therapeutic target for patients with these tumors, who otherwise have limited treatment options.

PAs, the most common pediatric brain tumors, are WHO grade I neoplasms with a generally benign clinical course and thus have an excellent prognosis, underscoring the importance of accurately distinguishing PAs from other glial neoplasms [29]. Most PAs display single genomic alterations, leading to MAPK pathway activation, the most common being a tandem duplication at 7q34, which is fairly specific for PA and results in *KIAA1459::BRAF* fusion [21, 40]. Mitogen-activated protein kinase kinase (MEK) inhibitors appear to be effective therapies for *KIAA1459::BRAF*-driven pediatric low grade gliomas, reducing tumor growth and improving survival [34].

In-frame fusions of members of the fibroblast growth factor receptor and transforming acidic coiled-coil gene families (*FGFR::TACC*) generate potent oncogenes with constitutive kinase activity [45]. Chromosomal translocations creating *FGFR::TACC* gene fusions have been discovered in many cancer types, including pediatric gliomas [54], and FGFR tyrosine kinase inhibitors have demonstrated promising effects in cancer patients with tumors carrying these fusions [26]. This type of targeted therapy is especially promising because it can be more specific and have fewer side effects than traditional chemotherapy or radiation therapy.

Craniopharyngiomas are rare, benign brain tumors with potentially devastating clinical effects. While BRAF inhibitors could benefit patients with papillary craniopharyngiomas, targeted therapies for the adamantinomatous type remain in development [4]. Agents targeting WNT signaling may have a promising effect in adamantinomatous craniopharyngiomas; however, current potential off-target effects limit their use. Nevertheless, the discovery that the MAPK and EGFR pathways appear dysregulated in this tumor subtype has led to the identification of various therapeutic targets that have shown promise in clinical strategies [16].

Gene fusions involving RELA in ependymoma activate the NF-KB transcription pathway and drive tumorigenesis [38]. Interestingly, blocking the NF-KB pathway by proteasome inhibitors may have a beneficial therapeutic effect in the ependymoma RELA-fusion molecular subgroup, and marizomib is being investigated for this purpose (ClinicalTrials.gov Identifier: NCT03727841).

Subependymal giant cell tumors are well-known manifestations of tuberous sclerosis caused by alterations in *TSC1* or *TSC2*, and the pathogenic activation of mTOR leads to tumor development [29]. Therefore, mTOR inhibitors have the potential to reduce tumor growth. Pineoblastoma is a rare and highly aggressive childhood brain cancer. *DICER1* encodes a ribonuclease involved in microRNA processing, and mutations in this gene are a known predisposing factor for pineoblastoma [10]. Promising management options that upregulate Dicer or promote the read-through of premature stop codons to restore Dicer transcription and translation are expected for these patients [17, 41].

Furthermore, epigenetic targeting is currently an active research focus in drug development, and the inhibition of enzymatic activities involved in epigenetic silencing driven by histone deacetylases, DNA methyltransferases and EZH2 is the subject of clinical trials that may be of value for treating ATRT [35].

Finally, because of its high mutation rate and critical role in driving cancer, the targeting of mutant *TP53* is of high importance. Although multiple strategies to reverse the oncogenic effects of *TP53* have been explored, clinical trials testing these agents have been aborted or revealed that the agents lacked efficacy. Compounds reactivating mutant p53 to produce wild-type p53 tumor suppressor activities are under investigation, and improvement of their properties may allow their therapeutic use to target mutant p53 [2, 6].

All these examples reveal the power of NGS to identify a wide variety of actionable gene alterations. Since the number of prognostic and predictive oncologic genetic markers is steadily increasing, it is crucial that NGS methods are applied in the clinic and that sequencing results are considered in directing pediatric treatments for individual cancer patients. Furthermore, diagnosing germline tumor-predisposing conditions can be beneficial both to the patient and other family members, allowing the implementation of adequate cancer screening programs. Making the power of NGS available in healthcare will is a challenging task involving integrating the work of clinical specialists, sequencing and bioinformatics facilities, and cancer biologists; this process requires a dedicated infrastructure, as exemplified here by the BTB.

## Conclusions

Here, we present the BTB, a nonprofit nationwide organization focused on serving as an efficient research infrastructure for collecting tissue and generating deep genomic data from pediatric patients with solid tumors. Its primary objective is to improve our understanding of pediatric cancer biology, with the ultimate aim of improving outcomes for children with cancer. To test the potential implications of the BTB workflow, we analyzed NGS data generated from a pilot set of biobanked tumor and blood samples collected from patients with CNS tumors and managed to identify somatic and germline alterations with biological or clinical significance. Our findings suggest that the implemented BTB workflow includes standard operating procedures that achieve high-quality biospecimens, high-quality extracted DNA and high-quality genomic data, thus a workflow that holds a potential to be implemented also in a clinical setting. The overall combined sequencing and methylation results confirmed or complemented the pathological tumor diagnosis made by clinical pathologists in 96% of cases (79 of 82 tumors), and the findings from NGS data specifically revealed a broad spectrum of mutations in known or likely driver genes in most patients (68 of 79) that supported the pathological diagnosis or the methylation-based classification (Additional file 2: Table S1).

Furthermore, the identified relevant mutations were not only of somatic but also of germline origin. Among the latter, patients carrying constitutional driver mutations in *TP53*, *TSC2* and *DICER1* were identified. The discovery of germline mutations in cancer patients may have important implications for their treatment and follow-up strategies. For example, patients with germline *TP53* mutations may be at an increased risk of developing secondary malignancies, especially after radiation therapy, and patients with *TSC2* mutations may be more responsive to targeted therapies that inhibit the mTOR pathway. Identifying germline mutations can also help screen family members for their own risk of developing cancer.

We also observed a clear advantage of WGS over WES approaches. Recognizing smaller CNVs by WES was challenging and the identification of SV breakpoints to predict fusion genes, often occurring outside the protein-coding regions captured by WES, failed. Therefore, we currently regard WGS as the method of choice for detecting germline and somatic variants, providing a more complete picture of the genomic landscape, including SNVs, CNVs and SVs.

Since the establishment of the BTB as a combined omics and tissue biobank, the rate of sample collection has gradually increased. Since 2020, up to 90% of affected children in Sweden are registered at the BTB, over 1100 fresh frozen tissues with matched blood DNA are available for research, and corresponding genomic data are continuously being generated. In this development we have recognized the importance of maintaining ethical standards and adhering to national and international regulations for biobanking to ensure that the samples and data are used appropriately.

We expect that the accessibility of biological samples and data to approved research projects will foster further advances in basic tumor biology, and impact many aspects of clinical care of children with cancer by refining or clarifying diagnoses, identifying novel oncogenic drivers and therapeutic targets. The use of this more precise diagnostic information will facilitate pediatric treatment guideline and oncology practice updates, help physicians in directing patients towards optimal personalized treatments and/or clinical trials, and ultimately improve the outcomes of children with cancer.

## Supplementary Information

**Additional file 1.** Supplementary materials and methods information.

**Additional file 2: Table S1.** Summary of sequencing and methylation profiling results.

**Additional file 3: Table S2.** Summary of sequencing metrics.

**Additional file 4: Table S3.** Software versions used during bioinformatics analysis in Sarek.

**Additional file 5: Figure S1.** Copy number alterations detected in CNS tumors. Heatmap of CNVs across the series of tumors studied as determined by Control-FREEC. The x-axis shows the copy number status along the chromosomes (1-22, X, Y). Areas painted in blue or red indicate regions of loss or gain, respectively. A deeper color indicates a relatively higher copy number change, with the scale displayed to the right of the heatmap. CNVs are common events in pediatric brain tumors, and CNVs with clinical relevance were observed in several samples. Focal *SMARCB1* deletions were identified in ATRT-MYC subgroup tumors (P1315_108T, P2233_115T), while whole chr22 deletions were observed in ATRT-TYR subgroup tumors (P1148_109T, P4551_208T). In MBs, deletion of the whole chr6 was observed in a MB of the WNT class (P7708_102T), and 9q and/or 10q loss was observed in MBs of the SHH class (FAM2T, F0025678-0810, F0025679-0775, BTB0001T). PAs generally exhibit different types of aberrations that indicate activation of mitogen-activating protein kinase (MAPK) signaling. Most of the profiled PAs (15 of 20 tumors) displayed the typical tandem duplication at 7q34 that results in *KIAA1459::BRAF* in-frame fusion and leads to a constitutively active kinase lacking the BRAF autoregulatory domain. One PA (P4552_110T) presented with a *QKI::RAF1* fusion resulting from a translocation between chr3 and chr6; these rearrangements also led to small telomeric 3p gain and 6q deletions. Mutations and amplifications of tyrosine-kinase receptors, such as *PDGFRA* and *PDGFRB*, or their ligands, result in activation of the PI3K and Ras/Raf pathways and are of clinical relevance. Amplification of *PDGFRA *on chr4 was observed among GBM/PNET/embryonal tumor samples (P7708_127T, P7708_128T, P4551_209T, see also Additional file 2: Table S1). In agreement with previous knowledge, *PDGFRA* alterations were found in older children (P7708_127T and P4551_209T, both adolescents) and in combination with H3F3A K27M in one tumor and with *MET* amplification (chr7, 35 copies) in the other [25, 31]. MET signaling aberrations are found in many human malignancies, and *MET* amplification as well as *MET* fusions have been observed in pediatric GBMs [19]. Additional alterations, such as homozygous loss of *CDKN2A* (P1148_112T, P4551_218T, P2233_106T) and amplification of *CDK6* (P4551_211T, P4551_209T, P2233_101T), were also observed in this series. Regarding ependymomas, the methylation classifier assigned three of them as ependymoma posterior fossa group A (EP-PFA) and four as ependymoma RELA fusion (EP-RELA). The methylation class EP-PFA mainly comprises tumors with scarce copy number changes [38]. Driving molecular changes in this class currently unknown. Two of the tumors in this class presented a flat profile (P2233_113T and P7708_111T), and the third showed 17q gain and 22q loss (F0025588-0809). The EP-RELA class almost exclusively comprises with an oncogenic fusion between *C11ORF95* and *RELA*, a member of the NF kappa B signaling pathway [38]. CNVs on 11q13.1, involving *C11ORF95* and *RELA,* were observed in four cases (P1148_114T, P7708_114T, P7708_115T and P7708_201T). In one of the EP-RELA samples (P1148_114T), gain of 1q was also observed, an alteration associated with poor prognosis [22]. In a pineoblastoma (P2233_112T) classified by methylation profiling as pineoblastoma group B, we found various numeric whole chromosome changes, which are frequently seen in this methylation class, including gain of chromosomes 7, 9, 12, 13, 15, 17, 18, 19 and 20. Furthermore, chromothripsis affecting chr11 was detected in the sample (Additional file 6: Fig. S2). In an atypical choroid plexus papilloma, classified as choroid plexus papilloma subclass pediatric A (P7708_108T), several whole chromosome gains, including trisomy 7, 8, 12, 18 and 19, were observed. Numeric whole chromosome changes are frequently observed in this tumor class; however, additional characteristic molecular features are unknown (https://www.molecularneuropathology.org/mnp/). One investigated hypophysis adenoma (A: P7708_125T) was classified as pituitary adenoma somatotropin producing, densely granulated group B. Pituitary adenomas (STH producing, densely granulated) form two methylation classes, group A and B, with unknown clinical significance. While group A tumors often present with several numeric whole chromosome changes, group B tumors seldom show chromosomal imbalances. The tumor studied here presented a flat profile that fits the methylation classification.

**Additional file 6: Figure S2.** Overlaid tumor and matched normal copy number profiles for specific chromosomes in selected cases, shown in black and pink, respectively. **A** PA diagnosed tumor (P4551_219T) for which methylation profiling could not find a matching class. Multiple SVs (at least 35), leading to numerous deletions and duplications, were present on chr22. Interestingly, an oncogenic fusion *EWSR1::PATZ1* was created as a result of these rearrangements (Table 1, Additional file 2: Table S1. This fusion has not been described in PA; nevertheless, it has recently been proposed that this fusion might define a new type of glioneuronal tumor [43]. **B**, **C** Focal 8p11.23-p11.22 duplication combined with inversion seen in a histopathologically diagnosed anaplastic PA (B: P4551_218T) and in an oligodendroglioma (C: P7708_105T), leading to *FGFR1::TACC1* fusion transcripts, Table 1, Additional file 2: Table S1. **(D-E)** A SHH-MB (FAM2T) displaying novel high focal copy number amplicons involving *BORCS5 *and *LMO3* (D) and a focal homozygous deletion of *SUFU* on chr10 (**E**). The amplicons encompassing *BORCS5 *and *LMO3* on 12p13.2 and 12p12.3 were present in ~65 DNA copies and may lead to the formation of a *LMO3::BORCS5* fusion. Fusions between these two genes have not been reported in MB but are seen in Ewing sarcoma, with *LMO3* acting as the driving oncogene. This patient also carries a pathogenic germline splice donor mutation in *TP53*, known to predispose to [15, 51], that was followed by somatic loss of the wild-type allele through LOH, Additional file 2: Table S1. **F** A novel amplicon on chr20 encompassing the *PLAGL2* and *POFUT1* genes in a MB sample (P4551_210T) that could not be classified by methylation arrays. *PLAGL2* has been proposed as an oncogene in gliomas [55], and *PLAGL2* and *POFUT1* collaboratively promote tumorigenesis in colorectal cancer by maintaining stemness [28]. **G **SVs in chr11 in an EP-RELA sample (P7708_114T) that led to *C11ORF95::RELA* fusion. **H**–**I **Chr19 profiles in two ETMRs (P4551_201T and P7708_101T), presenting the typical 19q13.42 focal amplification. **J **Chromothripsis affecting chr11 detected in one pineoblastoma group B (P2233_112T); over 230 SVs were observed.

**Additional file 7: Figure S3. **Whole genome copy number profiles for selected samples with multiple structural variants. The tumor copy number status is indicated in different colors according to a scale (0 to 4) shown to the right in the figure. The copy number profile for the paired normal sample is shown in gray below the tumor track. **A **Highly rearranged genome profile in a tumor diagnosed as PNET (P2233_101T) and classified by methylation profiling as GBM IDH wild-type (though with lower score, 0.61). *TP53* inactivation due to a somatic splice donor mutation plus LOH was observed. Numerous CNVs affecting almost all chromosomes and several focal amplifications were also detected, including amplification of *EGFR* on 7p11.2, *KRAS* on 12p12.1 (~70 DNA copies) and a nearby duplication (> 50 DNA copies) creating a *PLEKHA5::PIK3C2G* fusion (Table 1, Additional file 2: Table S1). **B** A GBM (P2233_118T) that could not be assigned to a specific class by the methylation classifier showed many SVs, including numerous focal deletions within chrX, focal duplications and deletions on chr15, as well as a focal *RB1* homozygous deletion (an extremely rare event in pediatric HGG). SV involving *NTRK3* and a predicted fusion involving *RERE::SLC2A5* were detected (Table 1, Additional file 2: Table S1). **C** An atypical meningioma (P2233_116T) displaying several copy number aberrations, including loss of chr22, which is a frequent event in pediatric meningiomas [32]. Multiple interchromosomal translocations were also observed on chr16, 19, 20 and 22, two of which involved *NF2* (chr22:29638300 T]CHR12:131449186] and chr22:29645023 [CHR3:86137311[T) and led to *NF2* transcript ablation. The tumor also displayed a focal amplification on 20q13.13, an event associated with progression and metastasis in several cancers. This locus encompasses, among others, *ZFAS1*, encoding a noncoding RNA that regulates the expression of genes involved in differentiation. An oncogenic role for ZFAS1 lncRNA has been described in head and neck squamous cell carcinomas [24]. **D** A relapsed rhabdoid meningioma (P2233_106T), for which the methylation classifier could not find a match. Rhabdoid meningioma is a rare, aggressive meningioma subtype. The sample presented with a highly rearranged genome with numerous SVs affecting almost all chromosomes, including deletion of chr22, encompassing *NF2*, an inversion on chr15 predicting a fusion between *GABRA5* and *NTRK3* and focal amplifications on chr5, several of them involving *RICTOR*. Focal homozygous deletions were also observed, one encompassing *CDKN2A* and another on 22q13.33, including *SHANK3*. Furthermore, the child carried a germline missense mutation in *TP53* chr17:7674230 C/T rs28934575 245/393 G/S, with loss of the wild-type allele in the tumor (see Table 1, Additional file 2: Table S1.

## Data Availability

The sequence data presented in this paper contains sensitive information that cannot be shared openly. The data will be shared under controlled access via FEGA Sweden node, which is hosted by the National Bioinformatics Infrastructure Sweden (NBIS) at SciLifeLab, once this repository becomes operational. The datasets will then be findable through the European Genome-phenome Archive web portal (https://ega-archive.org). Information about the datasets included in this study is available at SciLifeLab Data Repository, DOI, https://figshare.scilifelab.se/articles/dataset/The_Swedish_Childhood_Tumor_Biobank_Systematic_collection_and_molecular_characterization_of_all_pediatric_CNS_and_other_solid_tumors_in_Sweden/22249933 .

## References

[1] Amatu A, Sartore-Bianchi A, Siena S (2016). NTRK gene fusions as novel targets of cancer therapy across multiple tumour types. ESMO Open.

[2] Blandino G, Di Agostino S (2018). New therapeutic strategies to treat human cancers expressing mutant p53 proteins. J Exp Clin Cancer Res.

[3] Boeva V, Popova T, Bleakley K, Chiche P, Cappo J, Schleiermacher G, Janoueix-Lerosey I, Delattre O, Barillot E (2012). Control-FREEC: a tool for assessing copy number and allelic content using next-generation sequencing data. Bioinformatics.

[4] Brastianos PK, Taylor-Weiner A, Manley PE, Jones RT, Dias-Santagata D, Thorner AR, Lawrence MS, Rodriguez FJ, Bernardo LA, Schubert L (2014). Exome sequencing identifies BRAF mutations in papillary craniopharyngiomas. Nat Genet.

[5] Capper D, Jones DTW, Sill M, Hovestadt V, Schrimpf D, Sturm D, Koelsche C, Sahm F, Chavez L, Reuss DE (2018). DNA methylation-based classification of central nervous system tumours. Nature.

[6] Chakravarty D, Gao JJ, Phillips S, Kundra R, Zhang HX, Wang JJ, Rudolph JE, Yaeger R, Soumerai T, Nissan MH (2017). OncoKB: a precision oncology knowledge base. Jco Precis Oncol.

[7] Chen X, Schulz-Trieglaff O, Shaw R, Barnes B, Schlesinger F, Kallberg M, Cox AJ, Kruglyak S, Saunders CT (2016). Manta: rapid detection of structural variants and indels for germline and cancer sequencing applications. Bioinformatics.

[8] Cingolani P, Platts A, le Wang L, Coon M, Nguyen T, Wang L, Land SJ, Lu X, Ruden DM (2012). A program for annotating and predicting the effects of single nucleotide polymorphisms, SnpEff: SNPs in the genome of *Drosophila melanogaster* strain w1118; iso-2; iso-3. Fly.

[9] Consortium ITP-CAoWG (2020). Pan-cancer analysis of whole genomes. Nature.

[10] de Kock L, Sabbaghian N, Druker H, Weber E, Hamel N, Miller S, Choong CS, Gottardo NG, Kees UR, Rednam SP (2014). Germ-line and somatic DICER1 mutations in pineoblastoma. Acta Neuropathol.

[11] Ewels P, Magnusson M, Lundin S, Kaller M (2016). MultiQC: summarize analysis results for multiple tools and samples in a single report. Bioinformatics.

[12] Gambella A, Senetta R, Collemi G, Vallero SG, Monticelli M, Cofano F, Zeppa P, Garbossa D, Pellerino A, Ruda R (2020). NTRK fusions in central nervous system tumors: a rare, but worthy target. Int J Mol Sci.

[13] Garcia M, Juhos S, Larsson M, Olason PI, Martin M, Eisfeldt J, DiLorenzo S, Sandgren J, De Diaz ST, Ewels P (2020). Sarek: a portable workflow for whole-genome sequencing analysis of germline and somatic variants. F1000Res.

[14] Grill J, Le Teuff G, Nysom K, Blomgren K, Hargrave D, McCowage G, Bautista F, van Vuurden D, Dangouloff-Ros V, Puget S (2019). Biological medicine for diffuse intrinsic pontine gliomas eradication (biomede): results of the three-arm biomarker-driven randomized trial in the first 230 patients from Europe and Australia. Neuro Oncol.

[15] Grobner SN, Worst BC, Weischenfeldt J, Buchhalter I, Kleinheinz K, Rudneva VA, Johann PD, Balasubramanian GP, Segura-Wang M, Brabetz S (2018). The landscape of genomic alterations across childhood cancers. Nature.

[16] Hengartner AC, Prince E, Vijmasi T, Hankinson TC (2020). Adamantinomatous craniopharyngioma: moving toward targeted therapies. Neurosurg Focus.

[17] Hooten NN, Martin-Montalvo A, Dluzen DF, Zhang YQ, Bernier M, Zonderman AB, Becker KG, Gorospe M, de Cabo R, Evans MK (2016). Metformin-mediated increase in DICER1 regulates microRNA expression and cellular senescence. Aging Cell.

[18] Hussain I, Eloy JA, Carmel PW, Liu JK (2013). Molecular oncogenesis of craniopharyngioma: current and future strategies for the development of targeted therapies. J Neurosurg.

[19] International Cancer Genome Consortium PedBrain Tumor P (2016). Recurrent MET fusion genes represent a drug target in pediatric glioblastoma. Nat Med.

[20] Johnson A, Severson E, Gay L, Vergilio JA, Elvin J, Suh J, Daniel S, Covert M, Frampton GM, Hsu S (2017). Comprehensive genomic profiling of 282 pediatric low- and high-grade gliomas reveals genomic drivers, tumor mutational burden, and hypermutation signatures. Oncologist.

[21] Jones DT, Kocialkowski S, Liu L, Pearson DM, Backlund LM, Ichimura K, Collins VP (2008). Tandem duplication producing a novel oncogenic BRAF fusion gene defines the majority of pilocytic astrocytomas. Cancer Res.

[22] Kilday JP, Mitra B, Domerg C, Ward J, Andreiuolo F, Osteso-Ibanez T, Mauguen A, Varlet P, Le Deley MC, Lowe J (2012). Copy number gain of 1q25 predicts poor progression-free survival for pediatric intracranial ependymomas and enables patient risk stratification: a prospective European clinical trial cohort analysis on behalf of the children’s cancer leukaemia group (CCLG), societe Francaise d’oncologie pediatrique (SFOP), and international society for pediatric oncology (SIOP). Clin Cancer Res.

[23] Kim S, Scheffler K, Halpern AL, Bekritsky MA, Noh E, Kallberg M, Chen X, Kim Y, Beyter D, Krusche P (2018). Strelka2: fast and accurate calling of germline and somatic variants. Nat Methods.

[24] Kolenda T, Guglas K, Kopczynska M, Teresiak A, Blizniak R, Mackiewicz A, Lamperska K, Mackiewicz J (2019). Oncogenic role of ZFAS1 lncRNA in head and neck squamous cell carcinomas. Cells-Basel.

[25] Koschmann C, Zamler D, MacKay A, Robinson D, Wu YM, Doherty R, Marini B, Tran D, Garton H, Muraszko K (2016). Characterizing and targeting PDGFRA alterations in pediatric high-grade glioma. Oncotarget.

[26] Lasorella A, Sanson M, Iavarone A (2017). FGFR-TACC gene fusions in human glioma. Neuro Oncol.

[27] Lawrence MS, Stojanov P, Polak P, Kryukov GV, Cibulskis K, Sivachenko A, Carter SL, Stewart C, Mermel CH, Roberts SA (2013). Mutational heterogeneity in cancer and the search for new cancer-associated genes. Nature.

[28] Li DJ, Lin CW, Li NP, Du YH, Yang CX, Bai Y, Feng ZC, Su C, Wu RL, Song SL (2019). PLAGL2 and POFUT1 are regulated by an evolutionarily conserved bidirectional promoter and are collaboratively involved in colorectal cancer by maintaining stemness. EBioMedicine.

[29] Louis DN, Perry A, Wesseling P, Brat DJ, Cree IA, Figarella-Branger D, Hawkins C, Ng HK, Pfister SM, Reifenberger G (2021). The 2021 WHO classification of tumors of the central nervous system: a summary. Neuro Oncol.

[30] Ma X, Liu Y, Liu Y, Alexandrov LB, Edmonson MN, Gawad C, Zhou X, Li Y, Rusch MC, Easton J (2018). Pan-cancer genome and transcriptome analyses of 1699 paediatric leukaemias and solid tumours. Nature.

[31] Mackay A, Burford A, Carvalho D, Izquierdo E, Fazal-Salom J, Taylor KR, Bjerke L, Clarke M, Vinci M, Nandhabalan M (2017). Integrated molecular meta-analysis of 1000 pediatric high-grade and diffuse intrinsic pontine glioma. Cancer Cell.

[32] Mawrin C, Kirches E, Sahm F, Bluecher C, Boekhoff S, Schuller U, Schittenhelm J, Snuderl M, Karajannis M, Perry A (2018). Pediatric meningiomas are characterized by distinct methylation profiles different from adult meningiomas. Neuro Oncol.

[33] McLaren W, Gil L, Hunt SE, Riat HS, Ritchie GR, Thormann A, Flicek P, Cunningham F (2016). The ensembl variant effect predictor. Genome Biol.

[34] Miklja Z, Pasternak A, Stallard S, Nicolaides T, Kline-Nunnally C, Cole B, Beroukhim R, Bandopadhayay P, Chi S, Ramkissoon SH (2019). Molecular profiling and targeted therapy in pediatric gliomas: review and consensus recommendations. Neuro Oncol.

[35] Nemes K, Fruhwald MC (2018). Emerging therapeutic targets for the treatment of malignant rhabdoid tumors. Expert Opin Ther Targets.

[36] Northcott PA, Buchhalter I, Morrissy AS, Hovestadt V, Weischenfeldt J, Ehrenberger T, Grobner S, Segura-Wang M, Zichner T, Rudneva VA (2017). The whole-genome landscape of medulloblastoma subtypes. Nature.

[37] Northcott PA, Shih DJ, Peacock J, Garzia L, Morrissy AS, Zichner T, Stutz AM, Korshunov A, Reimand J, Schumacher SE (2012). Subgroup-specific structural variation across 1000 medulloblastoma genomes. Nature.

[38] Pajtler KW, Witt H, Sill M, Jones DT, Hovestadt V, Kratochwil F, Wani K, Tatevossian R, Punchihewa C, Johann P (2015). Molecular classification of ependymal tumors across all cns compartments, histopathological grades, and age groups. Cancer Cell.

[39] Parsons DW, Roy A, Yang Y, Wang T, Scollon S, Bergstrom K, Kerstein RA, Gutierrez S, Petersen AK, Bavle A (2016). Diagnostic yield of clinical tumor and germline whole-exome sequencing for children with solid tumors. JAMA Oncol.

[40] Penman CL, Faulkner C, Lowis SP, Kurian KM (2015). Current understanding of braf alterations in diagnosis, prognosis, and therapeutic targeting in pediatric low-grade gliomas. Front Oncol.

[41] Robertson JC, Jorcyk CL, Oxford JT (2018). DICER1 syndrome: DICER1 mutations in rare cancers. Cancers.

[42] Robinson JT, Thorvaldsdottir H, Wenger AM, Zehir A, Mesirov JP (2017). Variant review with the integrative genomics viewer. Cancer Res.

[43] Siegfried A, Rousseau A, Maurage CA, Pericart S, Nicaise Y, Escudie F, Grand D, Delrieul A, Gomez-Brouchet A, Le Guellec S (2019). EWSR1-PATZ1 gene fusion may define a new glioneuronal tumor entity. Brain Pathol.

[44] Sievers P, Appay R, Schrimpf D, Stichel D, Reuss DE, Wefers AK, Reinhardt A, Coras R, Ruf VC, Schmid S (2019). Rosette-forming glioneuronal tumors share a distinct DNA methylation profile and mutations in FGFR1, with recurrent co-mutation of PIK3CA and NF1. Acta Neuropathol.

[45] Singh D, Chan JM, Zoppoli P, Niola F, Sullivan R, Castano A, Liu EM, Reichel J, Porrati P, Pellegatta S (2012). Transforming fusions of FGFR and TACC genes in human glioblastoma. Science.

[46] Steliarova-Foucher E, Colombet M, Ries LAG, Moreno F, Dolya A, Bray F, Hesseling P, Shin HY, Stiller CA, contributors I- (2017). International incidence of childhood cancer, 2001–10: a population-based registry study. Lancet Oncol.

[47] Sweet-Cordero EA, Biegel JA (2019). The genomic landscape of pediatric cancers: implications for diagnosis and treatment. Science.

[48] Tang M, Verhaak RGW (2016). A Molecular take on malignant rhabdoid tumors. Trends Cancer.

[49] Taylor MD, Northcott PA, Korshunov A, Remke M, Cho YJ, Clifford SC, Eberhart CG, Parsons DW, Rutkowski S, Gajjar A (2012). Molecular subgroups of medulloblastoma: the current consensus. Acta Neuropathol.

[50] Van Loo P, Nordgard SH, Lingjaerde OC, Russnes HG, Rye IH, Sun W, Weigman VJ, Marynen P, Zetterberg A, Naume B (2010). Allele-specific copy number analysis of tumors. Proc Natl Acad Sci U S A.

[51] Waszak SM, Northcott PA, Buchhalter I, Robinson GW, Sutter C, Groebner S, Grund KB, Brugieres L, Jones DTW, Pajtler KW (2018). Spectrum and prevalence of genetic predisposition in medulloblastoma: a retrospective genetic study and prospective validation in a clinical trial cohort. Lancet Oncol.

[52] Worst BC, van Tilburg CM, Balasubramanian GP, Fiesel P, Witt R, Freitag A, Boudalil M, Previti C, Wolf S, Schmidt S (2016). Next-generation personalised medicine for high-risk paediatric cancer patients—the INFORM pilot study. Eur J Cancer.

[53] Zhang J, Walsh MF, Wu G, Edmonson MN, Gruber TA, Easton J, Hedges D, Ma X, Zhou X, Yergeau DA (2015). Germline mutations in predisposition genes in pediatric cancer. N Engl J Med.

[54] Zhang J, Wu G, Miller CP, Tatevossian RG, Dalton JD, Tang B, Orisme W, Punchihewa C, Parker M, Qaddoumi I (2013). Whole-genome sequencing identifies genetic alterations in pediatric low-grade gliomas. Nat Genet.

[55] Zheng H, Ying H, Wiedemeyer R, Yan H, Quayle SN, Ivanova EV, Paik JH, Zhang H, Xiao Y, Perry SR (2010). PLAGL2 regulates Wnt signaling to impede differentiation in neural stem cells and gliomas. Cancer Cell.

