# Supplementary material for: The Swedish childhood tumor biobank: systematic collection and molecular characterization of all pediatric CNS and other solid tumors in Sweden
Source: J Transl Med. 2023 May 23;21:342. doi: 10.1186/s12967-023-04178-4 (PMC10204274; doi:10.1186/s12967-023-04178-4)
Supplement: Supplementary file 1 — Additional file 1. Supplementary materials and methods information. [file 12967_2023_4178_MOESM1_ESM.docx]

## *Supplementary data 1:*

*Sample workflow: Informed consent process, sample and data collection*

Biospecimens and deep genetic data from pediatric patients diagnosed with CNS and other solid tumors in Sweden are collected, processed and stored long-term at the BTB. A multidisciplinary team successfully maintains and develops this combined biobank. Depending on practice variations within the clinical sites where the samples are collected, either the oncologist or the surgeon introduces the patient or patient’s parents to the BTB project and invites their participation to donate biospecimens (leftover samples) obtained during clinical interventions and allow the generation and use of sensitive molecular data in medical research projects. Written informed consent is acquired from the affected child’s parents or legal guardians and the patient him or herself when appropriate prior to processing. Participants agree prospectively for samples, restricted health information and genomic data to be kept at the BTB to be used in ethically approved and qualified pediatric cancer research projects. Tissue samples are collected during surgery and immediately kept on ice until they are snap frozen (in liquid nitrogen or isopentane) or immersed in RNA-later (Invitrogen) in under 30 min to reduce ischemic time. For sampling viable cells, cell suspensions are prepared from fresh tumor tissue (pieces ~3x3 mm in size) and frozen in 10% DMSO, serum-free medium (in liquid nitrogen vapor phase, -150 °C). The sampling of viable cells is performed locally at the clinical sites’ facilities and only upon sufficient tissue availability. To avoid limitations in the intended research, the BTB focuses on collecting fresh frozen tissue, which provides much better-quality DNA and RNA than formalin-fixed paraffin-embedded (FFPE) tissue [1]. Paired blood from the affected child and his or her parents is also collected (EDTA tubes) and kept at 4 °C for no over 72 h before processing for DNA extraction; otherwise, it is stored directly at -80 °C [2]. Additional biospecimens, including FFPE tissue, needle aspirates and cerebrospinal fluid, are occasionally donated by patients and obtained through the normal clinical management pathways at the hospitals in accordance with the BTB’s ethical permit.

Samples are delivered to the BTB on dry ice in secure transport aluminum cases (ZARGES K470). Semiautomated nucleic acid extraction from tumor tissues and blood is routinely performed, and that material is also available for distribution. Tumors are histopathologically reassessed by specialized pathologists. For extraction, the frozen tissue is sectioned (30-100 sections, 20-30 µm) in a cryostat, and tumor cell content is estimated by a pathologist on hematoxylin and eosin (H&E)-stained slides (5-8 µm sections after every 300 µm of collected tissue). Isolation of genomic DNA and RNA is performed using the AllPrep DNA/RNA/Protein Mini kit (Qiagen) and blood DNA (minimum 1 ml) using the QIAamp® DNA Blood Midi/Maxi kit, vacuum protocol (Qiagen), according to the manufacturer’s instructions.

The quantification and qualification of extracted nucleic acids is conducted by spectrophotometric (ND-1000 spectrophotometer, NanoDrop Technologies, Wilmington, DE) and fluorescence-based (sQubit^®^ fluorimeter using Qubit™ dsDNA BR Assay Kit, Invitrogen^TM^) methods. The ratio of spectrophotometric readings, 260/280 nm, for approved samples is 1.7-1.9 for DNA and 1.9-2.1 for RNA. Quantity and quality assessment of genomic DNA and RNA is conducted on a 4200 TapeStation or 2100 Bioanalyzer (Agilent). A DNA integrity number (DIN) >7 and an RNA integrity number (RIN) >8 (scales 1 to 10) are required.

Library preparation and sequencing (WGS/WES and RNA sequencing) and DNA methylation profiling are ISO-accredited applications currently performed at the Genomic Production Center, SciLifeLab, Stockholm and Uppsala, Sweden. At the start of the project, we performed WES; however, since 2016, only WGS is performed. Sequencing was performed on a HiSeqX v2.5, RRID:SCR_016385, at the start of the project, and we now use an Illumina NovaSeq Sequencing System, RRID:SCR_016387. Libraries for WGS are currently prepared with Illumina TruSeq PCR-free or Illumina TruSeq Nano when a low amount of DNA is available. The current paired-end read length is 150 bp. Germline samples were sequenced to a minimum mean coverage of 30× and tumors to a minimum depth of 45× at the beginning of the project; this increased to 60× in 2019 and to 90× in 2021.

## To ensure traceability, all samples are registered into a Laboratory Information Management System from Stockholm Medical Biobank (LIMS SMB). Each aliquot of tissue and biofluid is labeled and tracked. Patients’ individual and clinical data are securely protected to guarantee confidentiality and correct handling of personal data. Inclusion of associated patient information is ethically and legally regulated, allowing the clinical diagnosis, age and sex to be saved alongside the samples. Supplementary clinically associated data relevant to research projects can be acquired through the National Quality Registry for Childhood Cancer (http://kvalitetsregister.se/). Generated genomic data are further pseudonymized and securely stored separately from associated clinical data. Tissue bank personnel follow in-house derived standard operating procedures for sample and data handling that are refined over

1 Coppola L, Cianflone A, Grimaldi AM, Incoronato M, Bevilacqua P, Messina F, Baselice S, Soricelli A, Mirabelli P, Salvatore M (2019) Biobanking in health care: evolution and future directions. J Transl Med 17: 172 Doi 10.1186/s12967-019-1922-3

2 Steinberg K, Beck J, Nickerson D, Garcia-Closas M, Gallagher M, Caggana M, Reid Y, Cosentino M, Ji J, Johnson Det al (2002) DNA banking for epidemiologic studies: a review of current practices. Epidemiology 13: 246-254 Doi 10.1097/00001648-200205000-00003
