# Supplementary figures and images for: The Swedish childhood tumor biobank: systematic collection and molecular characterization of all pediatric CNS and other solid tumors in Sweden
Source: J Transl Med. 2023 May 23;21:342. doi: 10.1186/s12967-023-04178-4 (PMC10204274; doi:10.1186/s12967-023-04178-4)

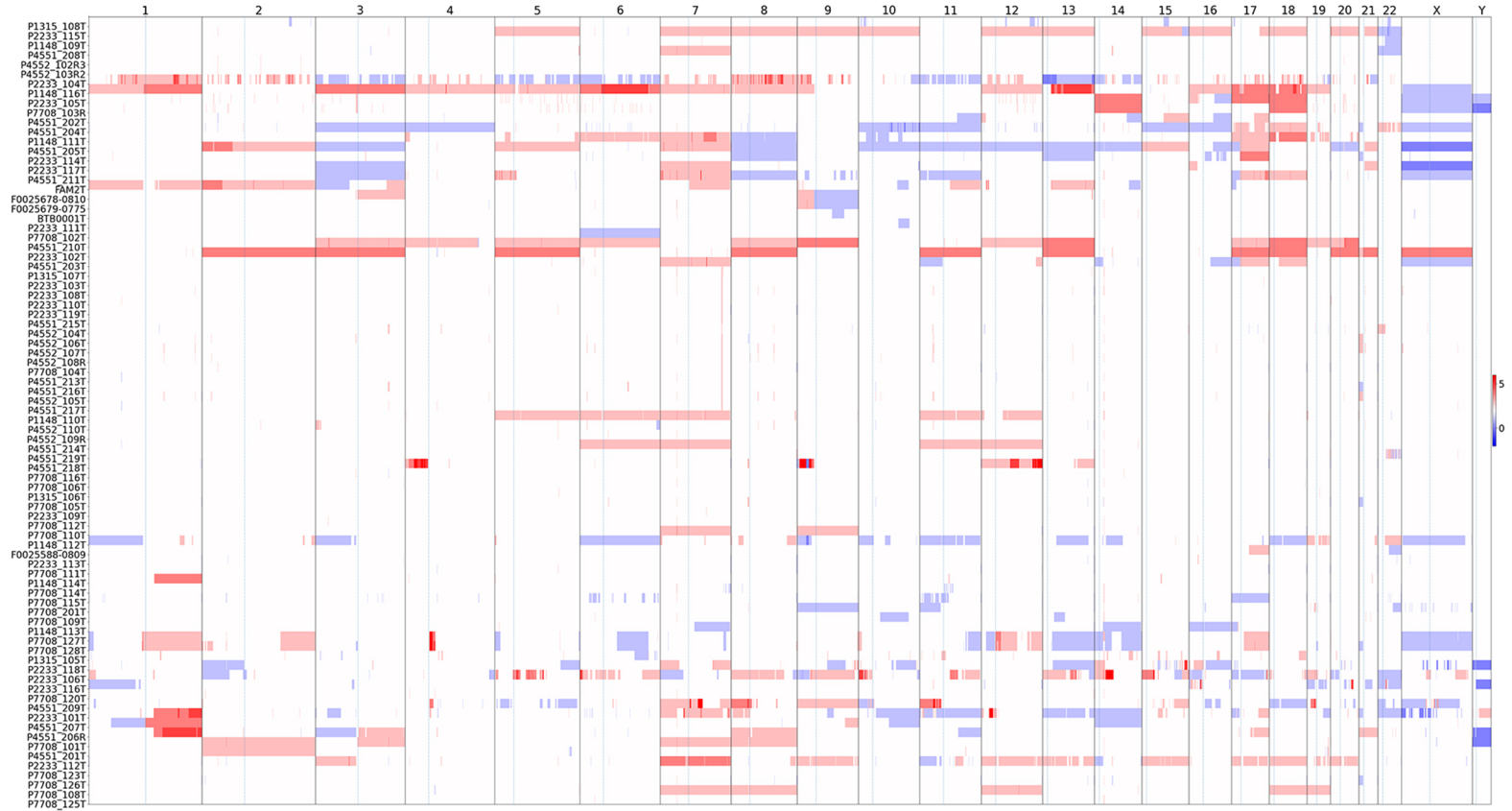

Supplement: Supplementary file 5 — Additional file 5: Figure S1. Copy number alterations detected in CNS tumors. Heatmap of CNVs across the series of tumors studied as determined by Control-FREEC. The x-axis shows the copy number status along the chromosomes (1-22, X, Y). Areas painted in blue or red indicate regions of loss or gain, respectively. A deeper color indicates a relatively higher copy number change, with the scale displayed to the right of the heatmap. CNVs are common events in pediatric brain tumors, and CNVs with clinical relevance were observed in several samples. Focal SMARCB1 deletions were identified in ATRT-MYC subgroup tumors (P1315_108T, P2233_115T), while whole chr22 deletions were observed in ATRT-TYR subgroup tumors (P1148_109T, P4551_208T). In MBs, deletion of the whole chr6 was observed in a MB of the WNT class (P7708_102T), and 9q and/or 10q loss was observed in MBs of the SHH class (FAM2T, F0025678-0810, F0025679-0775, BTB0001T). PAs generally exhibit different types of aberrations that indicate activation of mitogen-activating protein kinase (MAPK) signaling. Most of the profiled PAs (15 of 20 tumors) displayed the typical tandem duplication at 7q34 that results in KIAA1459::BRAF in-frame fusion and leads to a constitutively active kinase lacking the BRAF autoregulatory domain. One PA (P4552_110T) presented with a QKI::RAF1 fusion resulting from a translocation between chr3 and chr6; these rearrangements also led to small telomeric 3p gain and 6q deletions. Mutations and amplifications of tyrosine-kinase receptors, such as PDGFRA and PDGFRB, or their ligands, result in activation of the PI3K and Ras/Raf pathways and are of clinical relevance. Amplification of PDGFRA on chr4 was observed among GBM/PNET/embryonal tumor samples (P7708_127T, P7708_128T, P4551_209T, see also Additional file 2: Table S1). In agreement with previous knowledge, PDGFRA alterations were found in older children (P7708_127T and P4551_209T, both adolescents) and in combination with H3F3A K27M in one t [file 12967_2023_4178_MOESM5_ESM.pdf]
