# Supplementary material for: The Swedish childhood tumor biobank: systematic collection and molecular characterization of all pediatric CNS and other solid tumors in Sweden
Source: J Transl Med. 2023 May 23;21:342. doi: 10.1186/s12967-023-04178-4 (PMC10204274; doi:10.1186/s12967-023-04178-4)

**Figure S2**

**A:** P4551_219T - chr22 copy number profile displaying multiple SVs


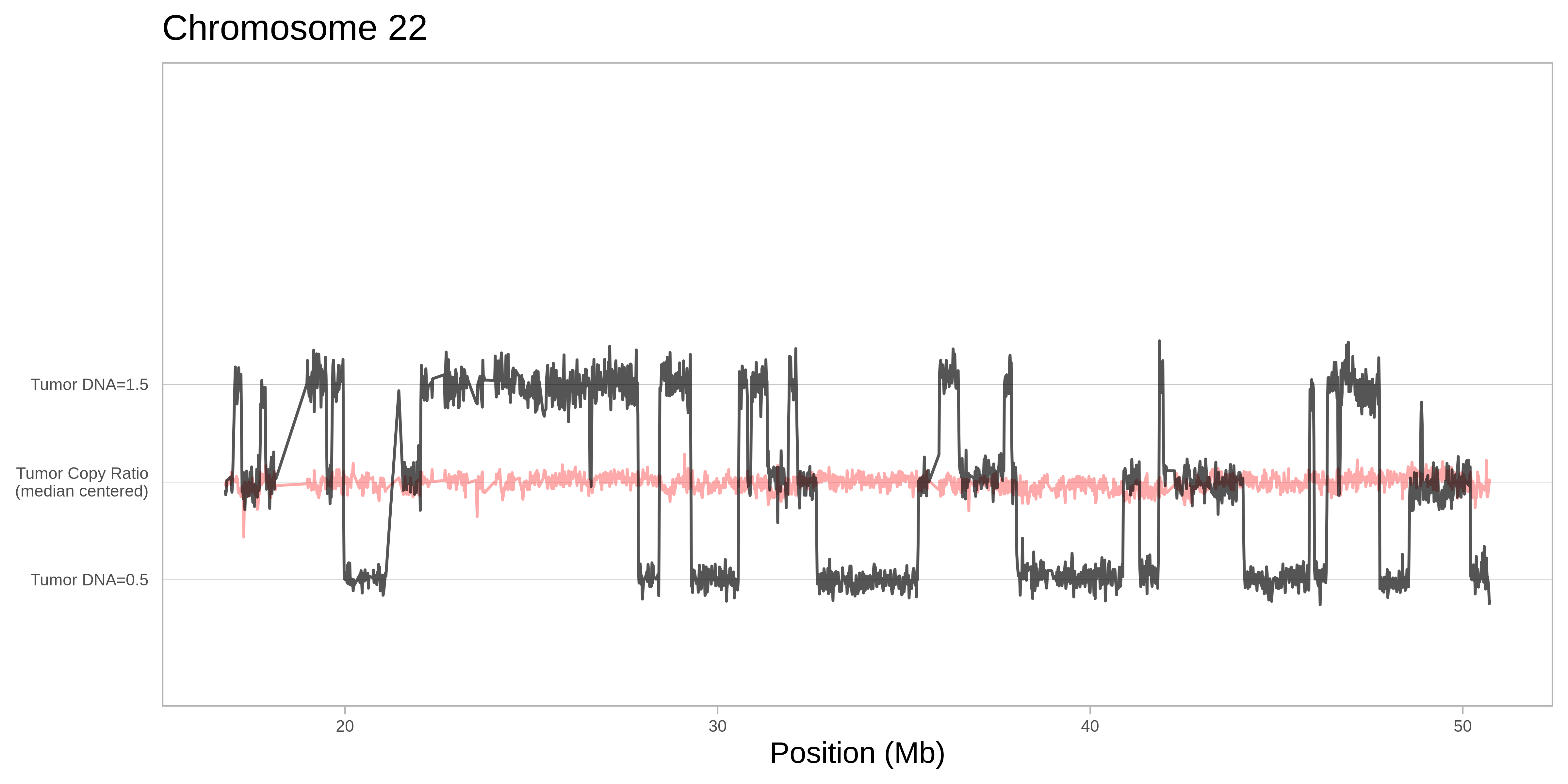


**B:** P4551_218T - chr8 copy number profile showing a focal duplication on 8p11.23-p11.22 forming *FGFR1-TACC1* fusion
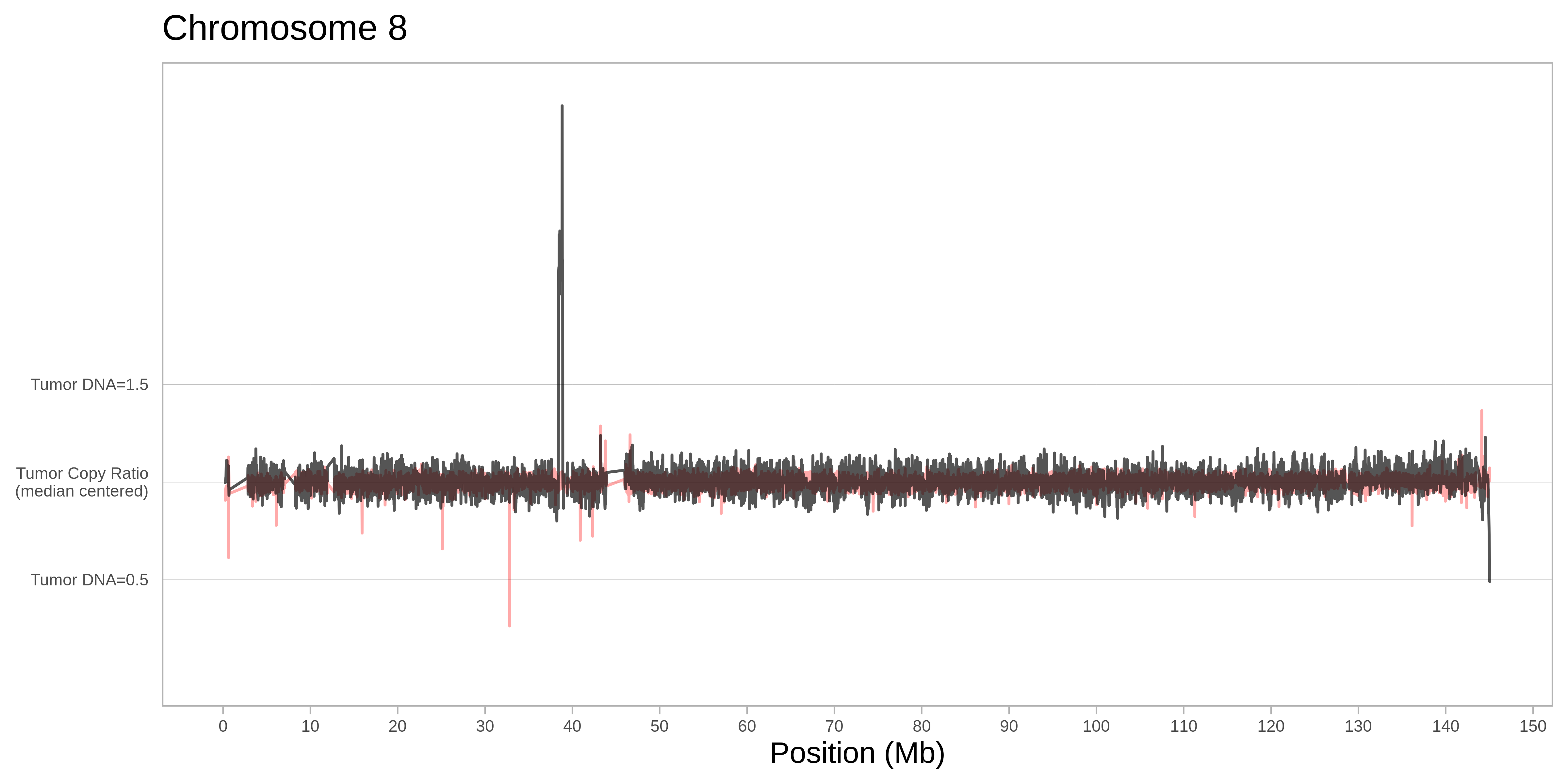


**C:** P7708_105T - chr8 copy number profile showing a focal duplication on 8p11.23-p11.22 forming *FGFR1-TACC1* fusion
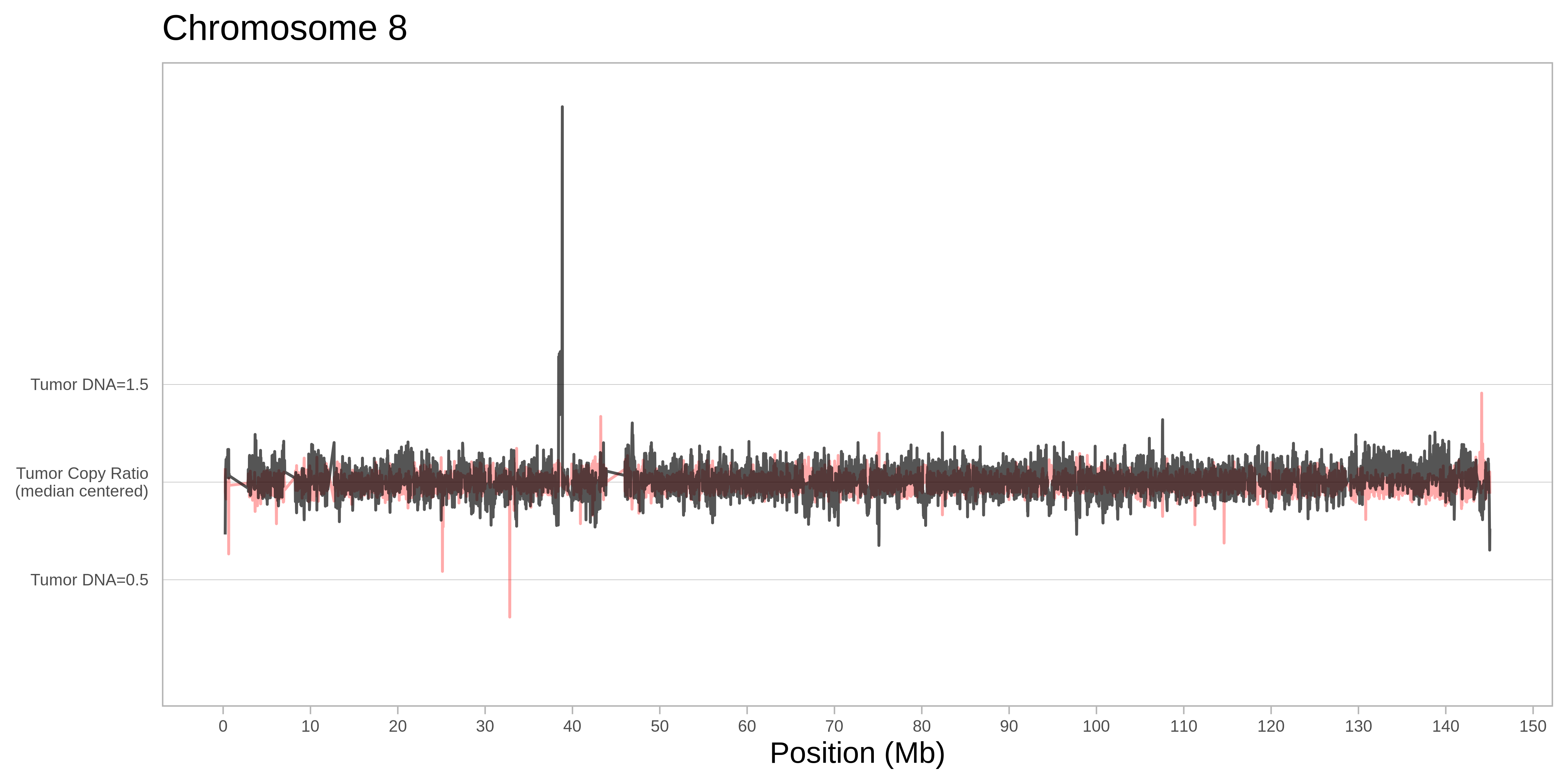


**D:** FAM2T – chr12 copy number profile showing focal amplification of *BORCS5* and *LMO3* in a MB SHH
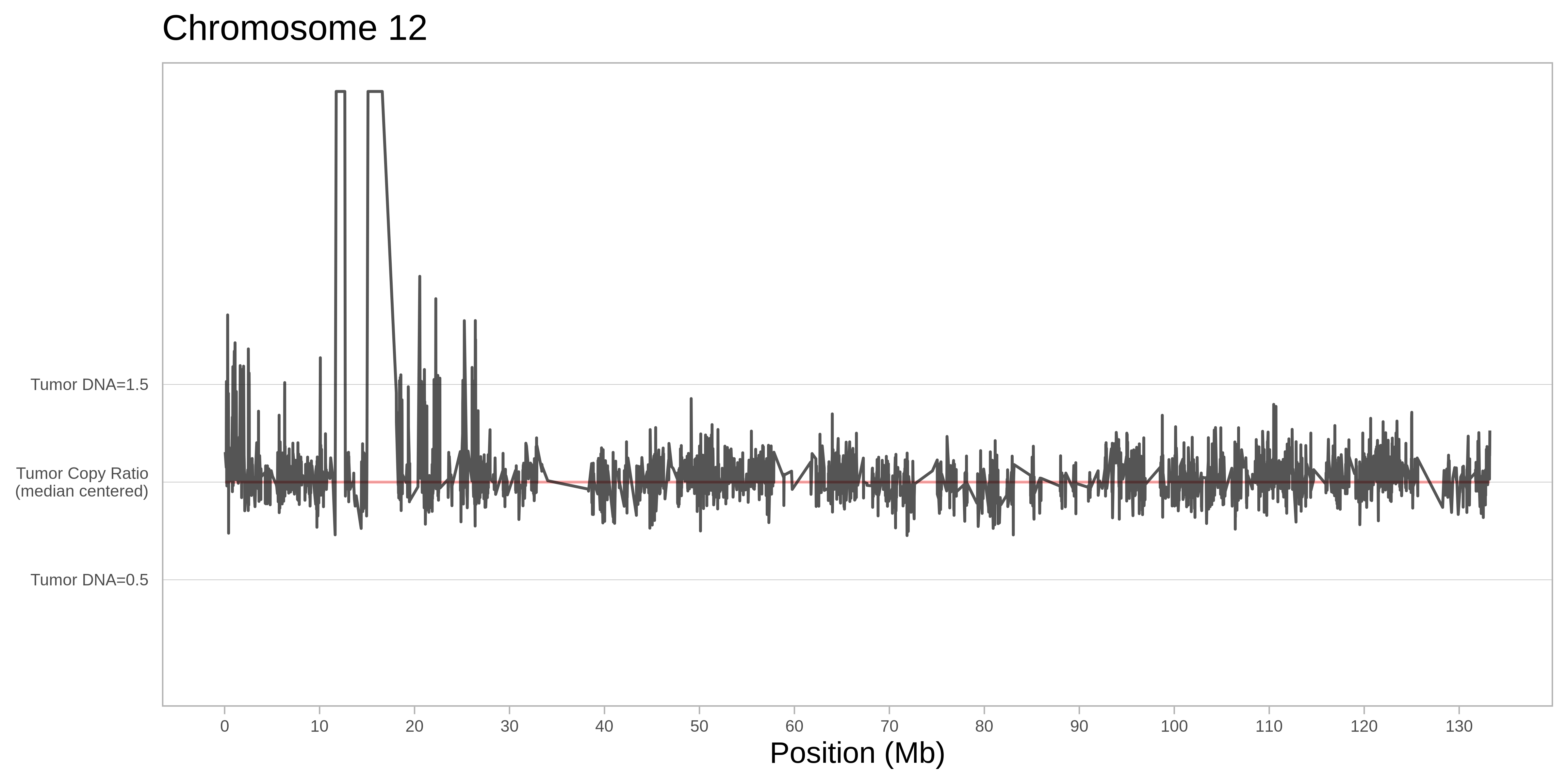


**E:** FAM2T - chr10 copy number profile showing a focal homozygous deletion of *SUFU* gene in a MB SHH
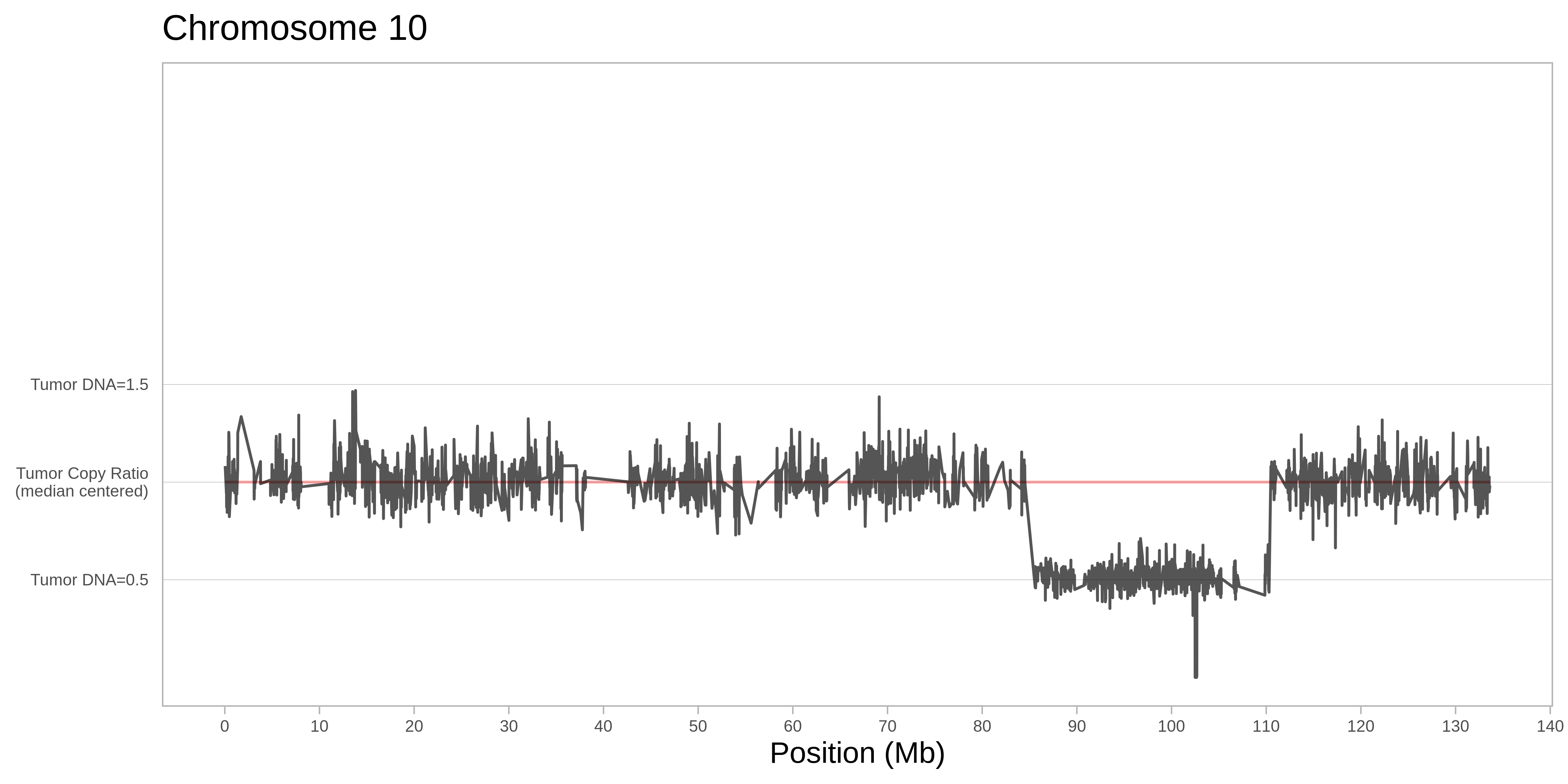


**F:** P4551_210T – chr20 copy number profile showing focal amplifications in a MB sample


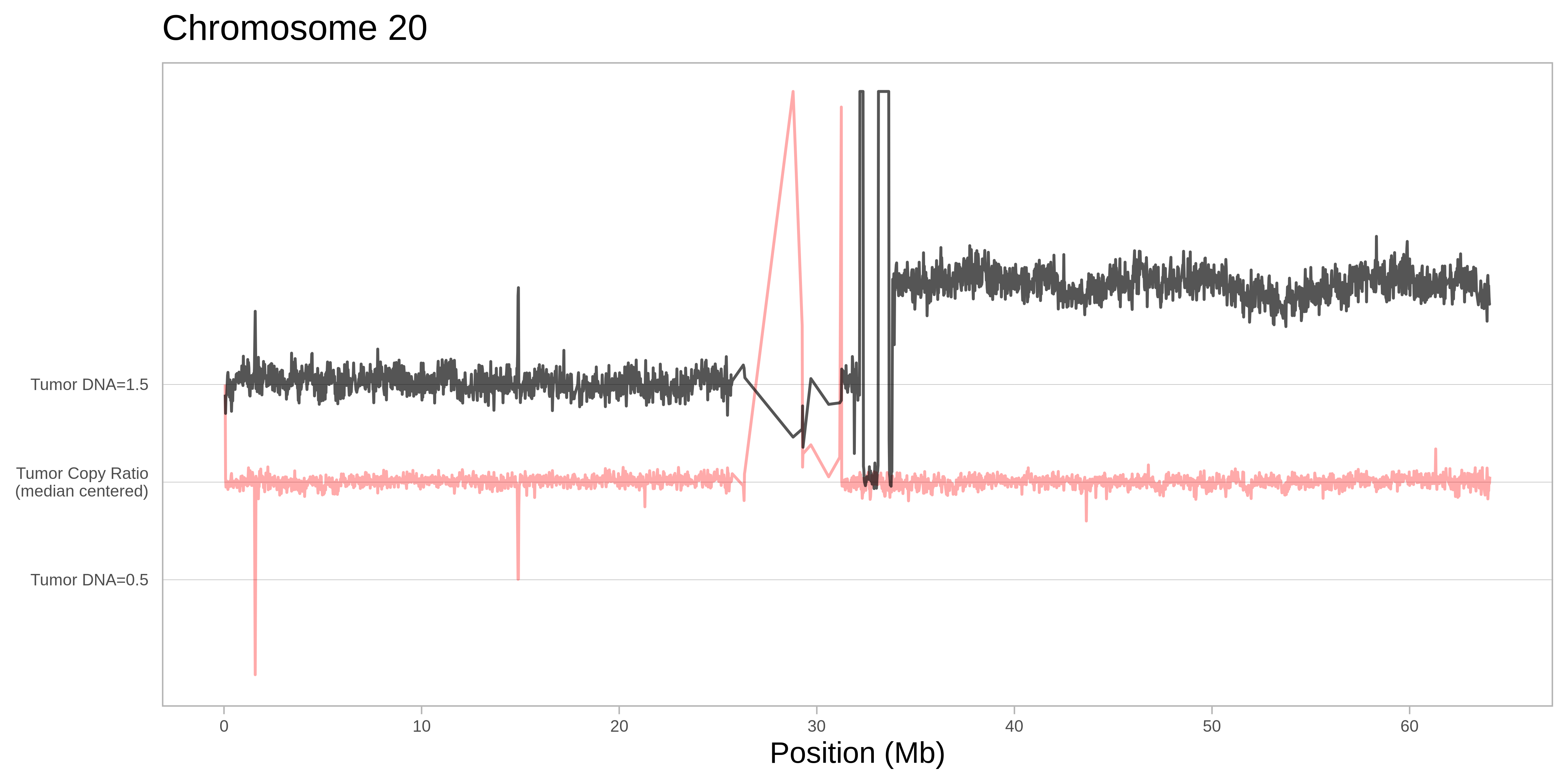


**G:** P7708_114T – chr11 copy number profile in a RELA class ependymoma


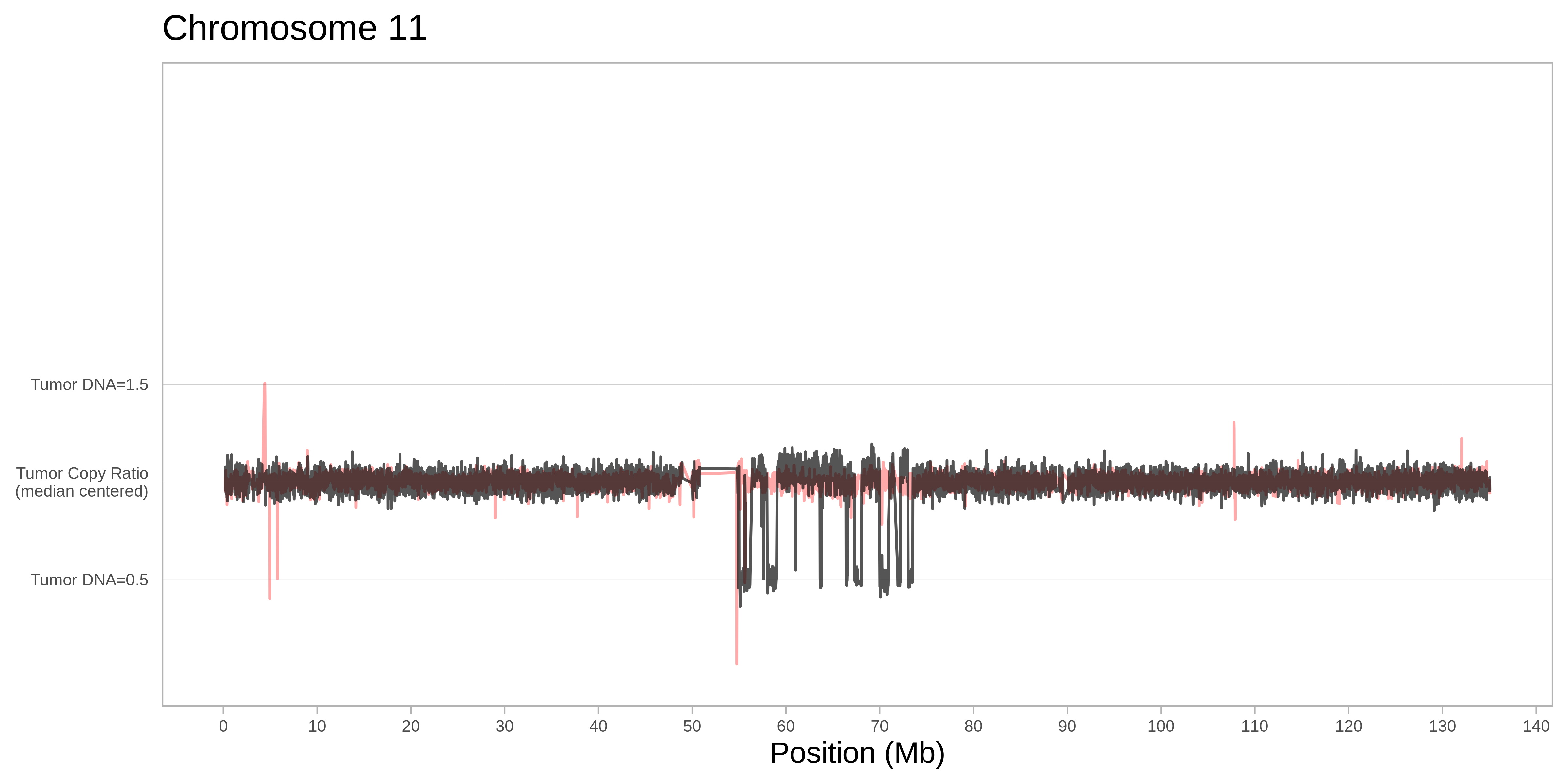


**H:** P4551_201T - chr19 copy number profile of an embryonal tumor with multilayered rosettes displaying amplification of the C19MC miRNA cluster on 19q13.42


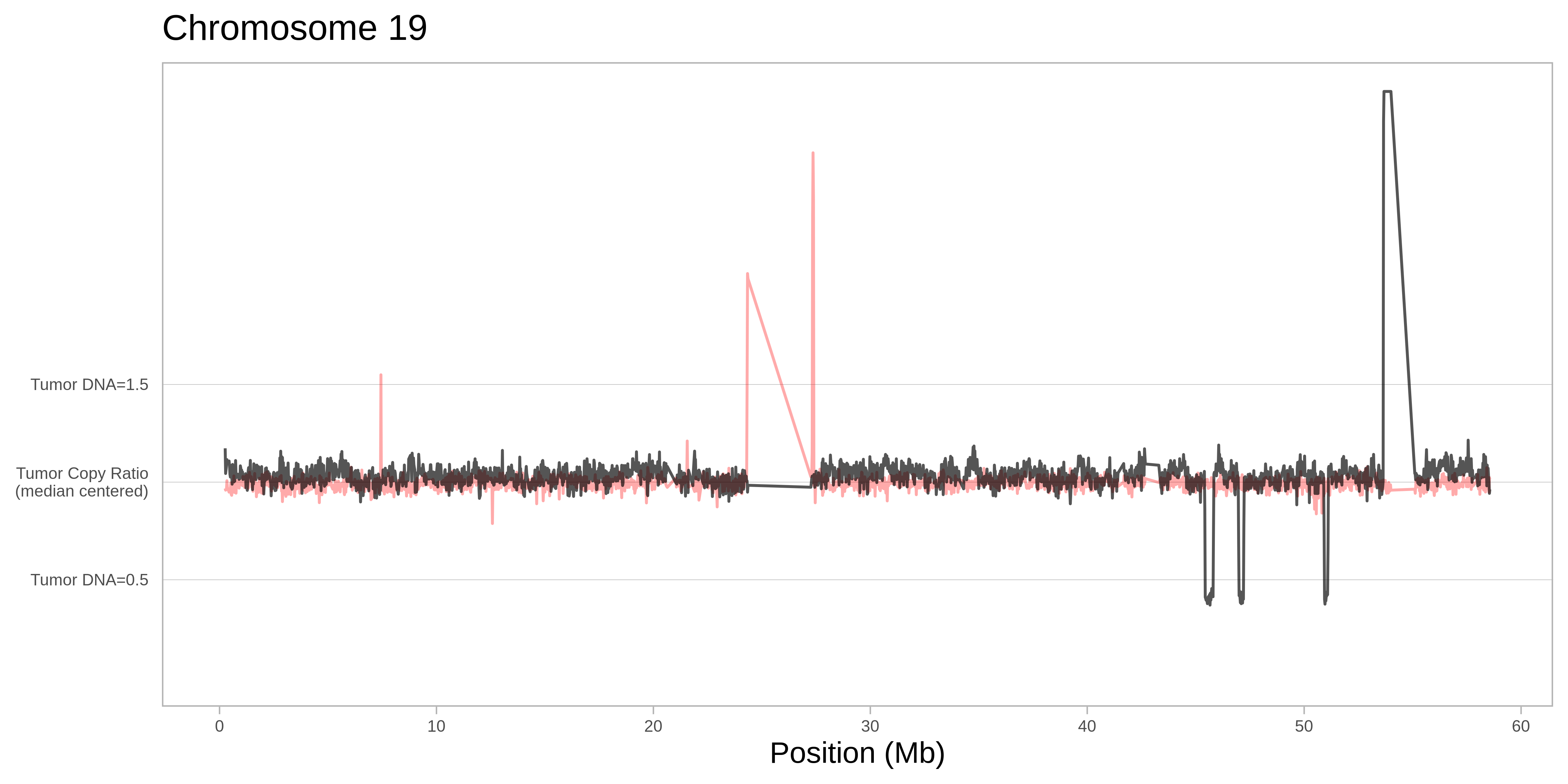


**I:** P7708_101T - chr19 copy number profile of an embryonal tumor with multilayered rosettes displaying amplification of the C19MC miRNA cluster on 19q13.42


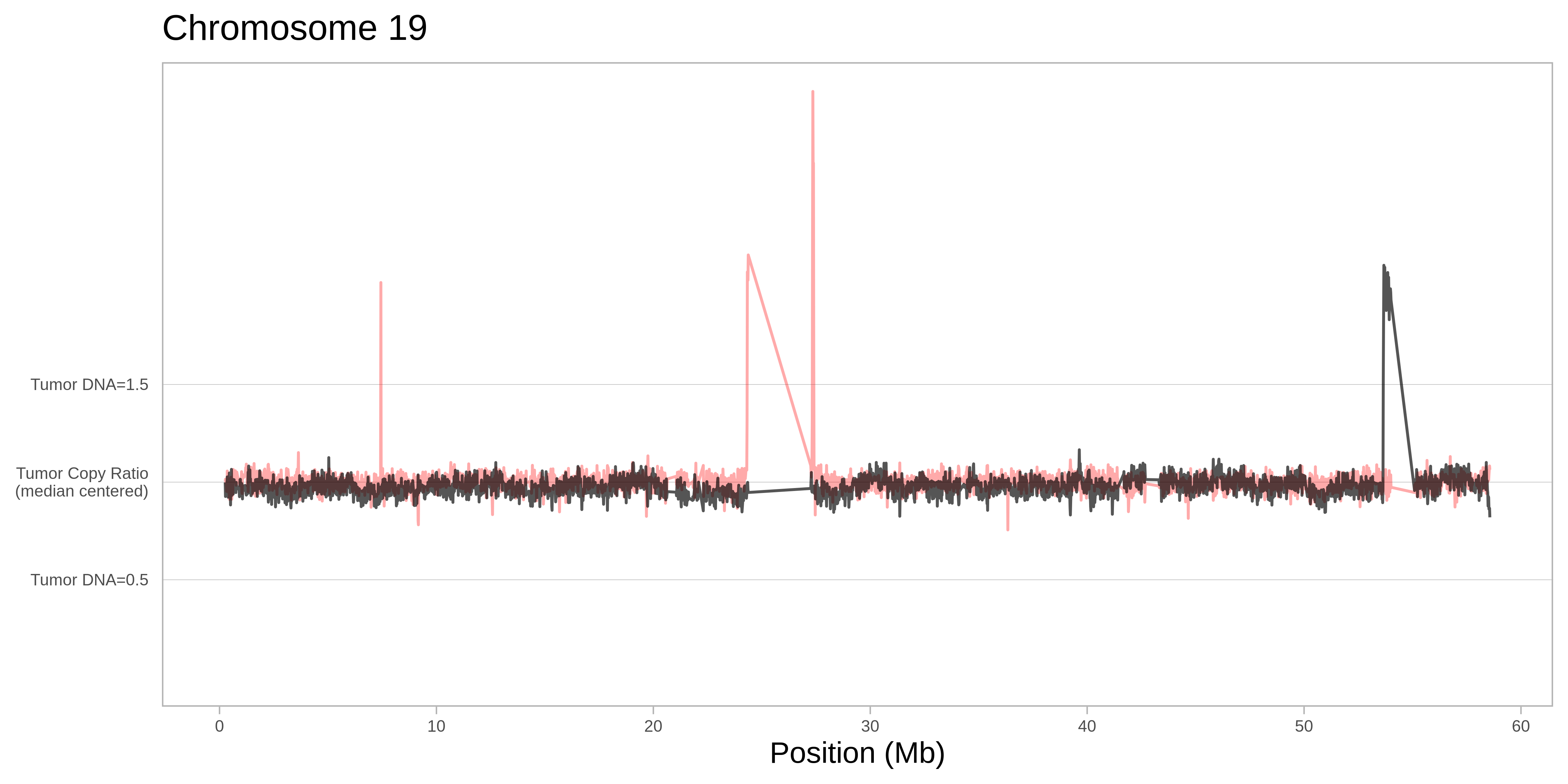


**J:** P2233_112T - chr11 copy number profile showing chromothripsis in a pineoblasatoma group B


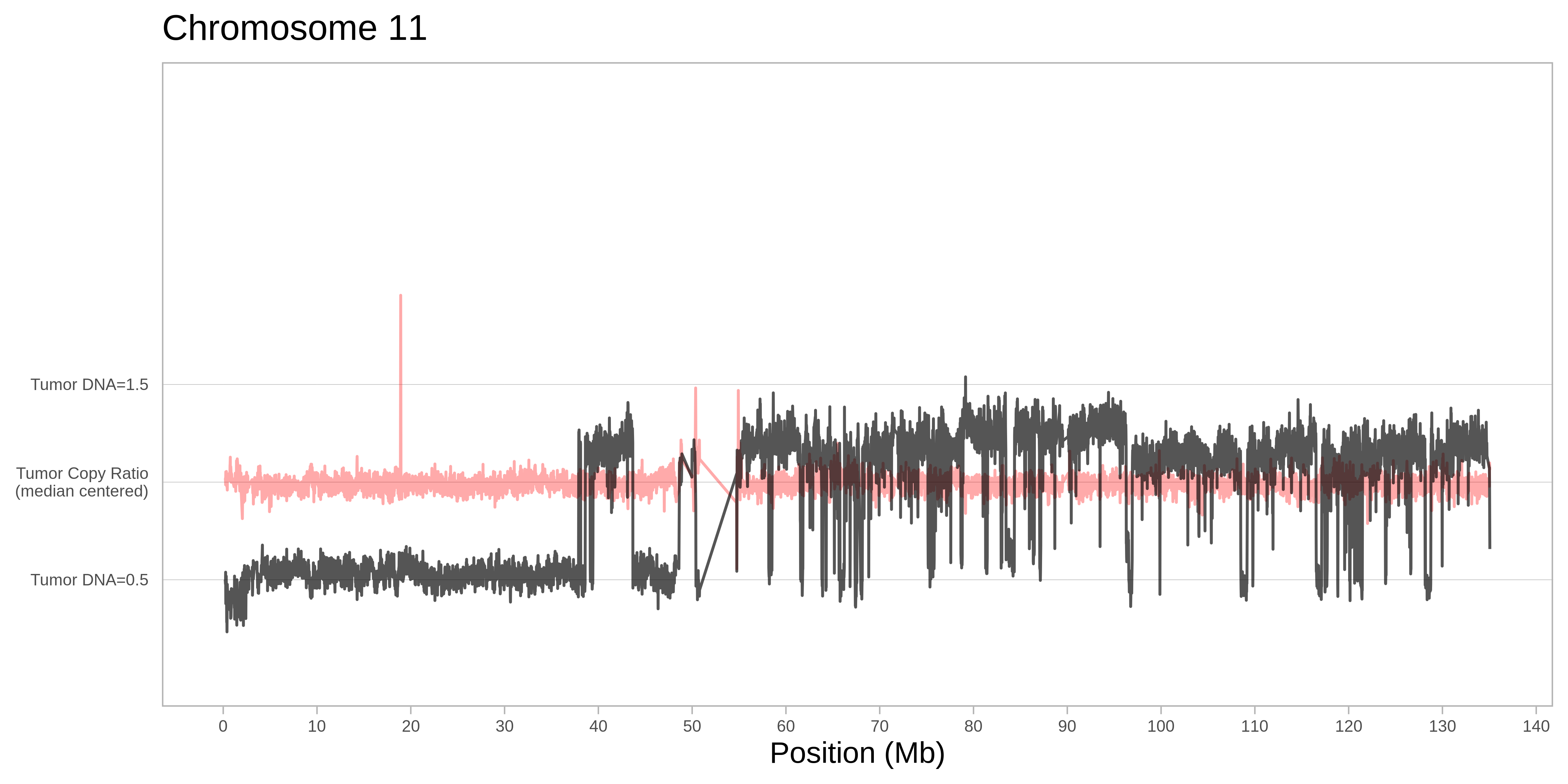

Supplement: Supplementary file 6 — Additional file 6: Figure S2. Overlaid tumor and matched normal copy number profiles for specific chromosomes in selected cases, shown in black and pink, respectively. A PA diagnosed tumor (P4551_219T) for which methylation profiling could not find a matching class. Multiple SVs (at least 35), leading to numerous deletions and duplications, were present on chr22. Interestingly, an oncogenic fusion EWSR1::PATZ1 was created as a result of these rearrangements (Table 1, Additional file 2: Table S1. This fusion has not been described in PA; nevertheless, it has recently been proposed that this fusion might define a new type of glioneuronal tumor [43]. B, C Focal 8p11.23-p11.22 duplication combined with inversion seen in a histopathologically diagnosed anaplastic PA (B: P4551_218T) and in an oligodendroglioma (C: P7708_105T), leading to FGFR1::TACC1 fusion transcripts, Table 1, Additional file 2: Table S1. (D-E) A SHH-MB (FAM2T) displaying novel high focal copy number amplicons involving BORCS5 and LMO3 (D) and a focal homozygous deletion of SUFU on chr10 (E). The amplicons encompassing BORCS5 and LMO3 on 12p13.2 and 12p12.3 were present in ~65 DNA copies and may lead to the formation of a LMO3::BORCS5 fusion. Fusions between these two genes have not been reported in MB but are seen in Ewing sarcoma, with LMO3 acting as the driving oncogene. This patient also carries a pathogenic germline splice donor mutation in TP53, known to predispose to [15, 51], that was followed by somatic loss of the wild-type allele through LOH, Additional file 2: Table S1. F A novel amplicon on chr20 encompassing the PLAGL2 and POFUT1 genes in a MB sample (P4551_210T) that could not be classified by methylation arrays. PLAGL2 has been proposed as an oncogene in gliomas [55], and PLAGL2 and POFUT1 collaboratively promote tumorigenesis in colorectal cancer by maintaining stemness [28]. G SVs in chr11 in an EP-RELA sample (P7708_114T) that led to C11ORF95::RELA fusion. H–I Chr19 profiles in two ET [file 12967_2023_4178_MOESM6_ESM.docx]
