# Supplementary material for: The Swedish childhood tumor biobank: systematic collection and molecular characterization of all pediatric CNS and other solid tumors in Sweden
Source: J Transl Med. 2023 May 23;21:342. doi: 10.1186/s12967-023-04178-4 (PMC10204274; doi:10.1186/s12967-023-04178-4)

**Figure S3**

**A:** P2233_101T - A CNS-PNET with highly rearranged genome profile.


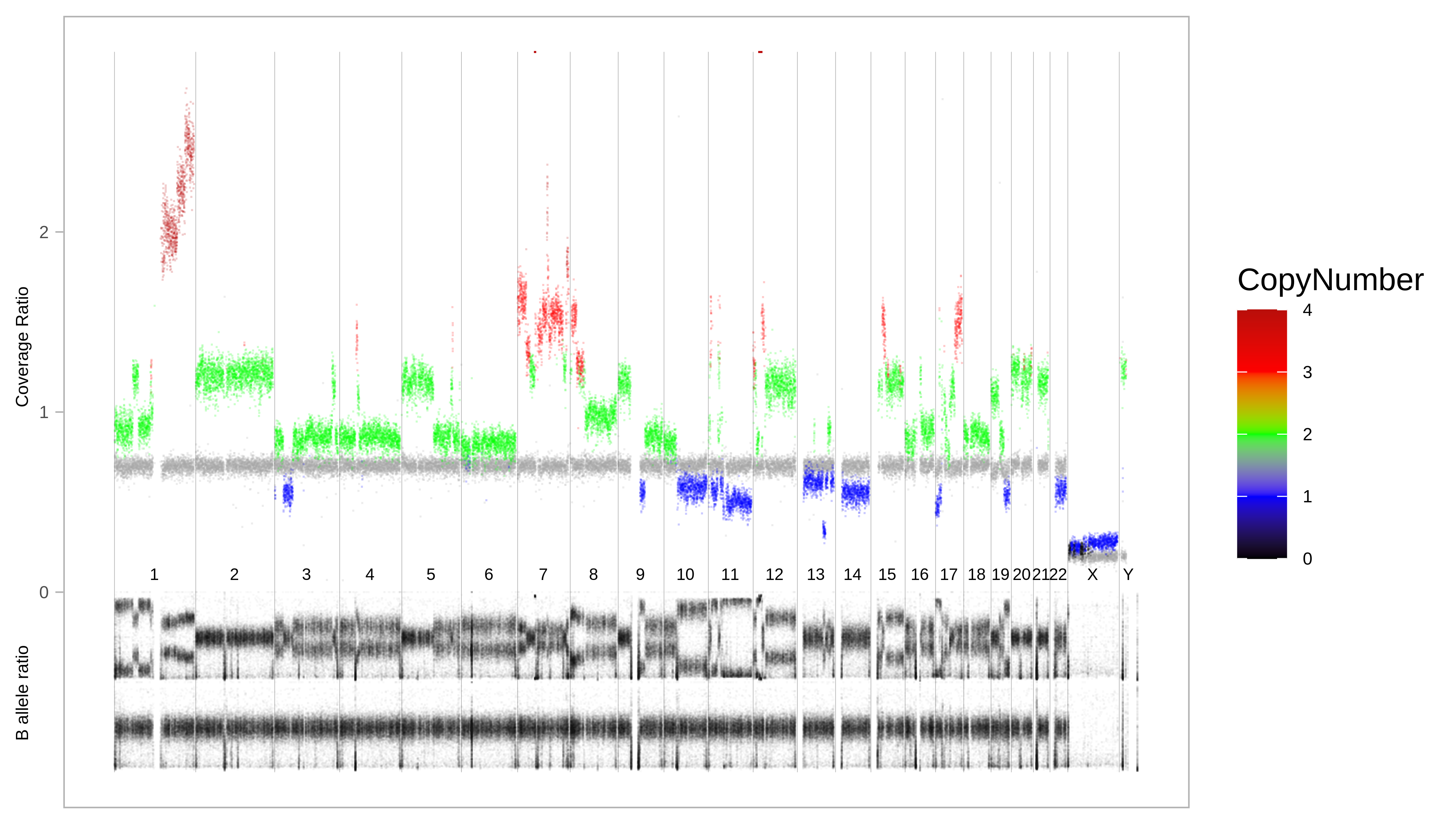


**B:** P2233_118T – Whole genome copy number profile in a glioblastoma showing various chromosomal rearrangements


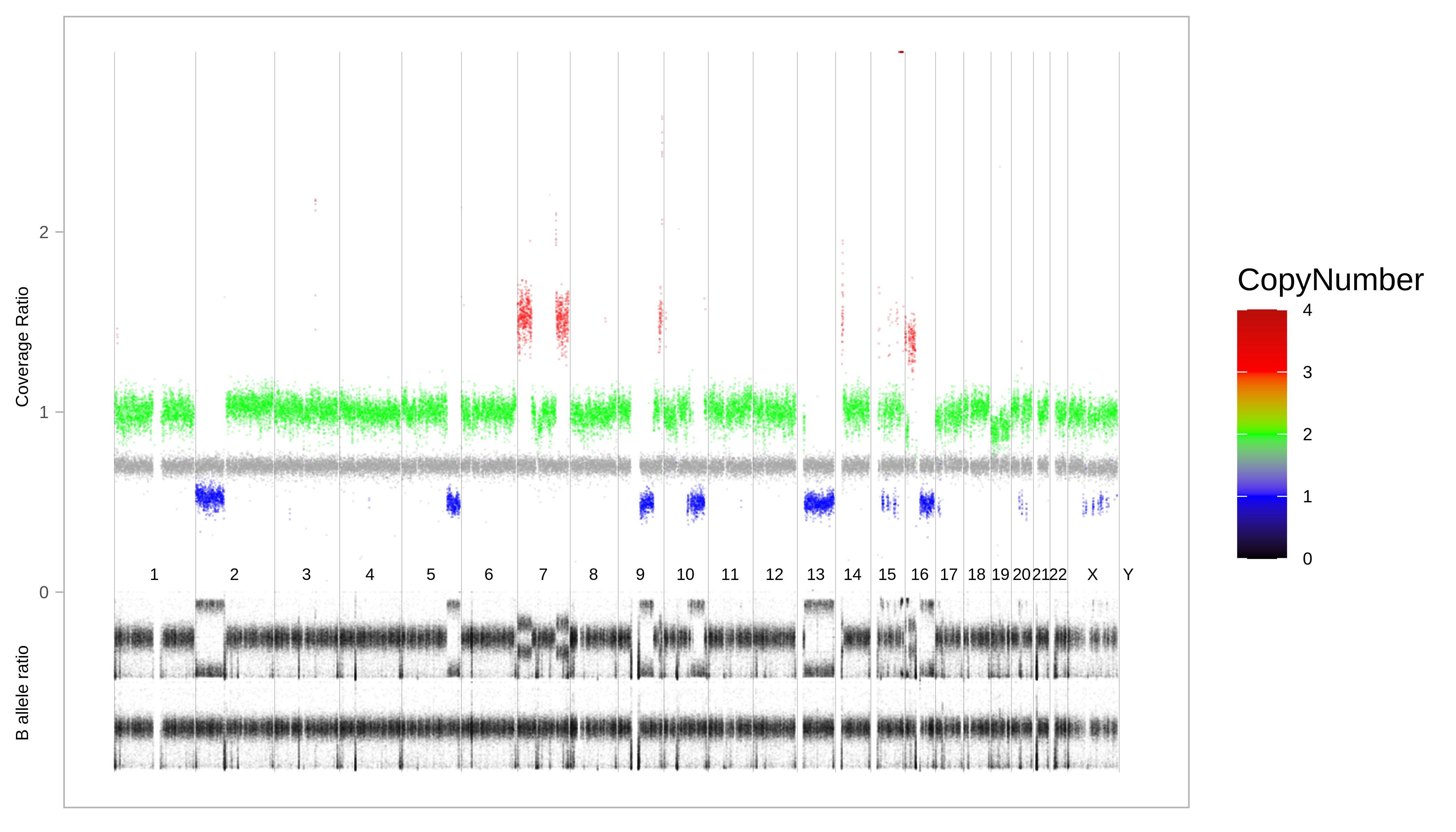


**C:** P2233_116T - Whole genome copy number profile of an atypical meningioma displaying several copy number aberrations


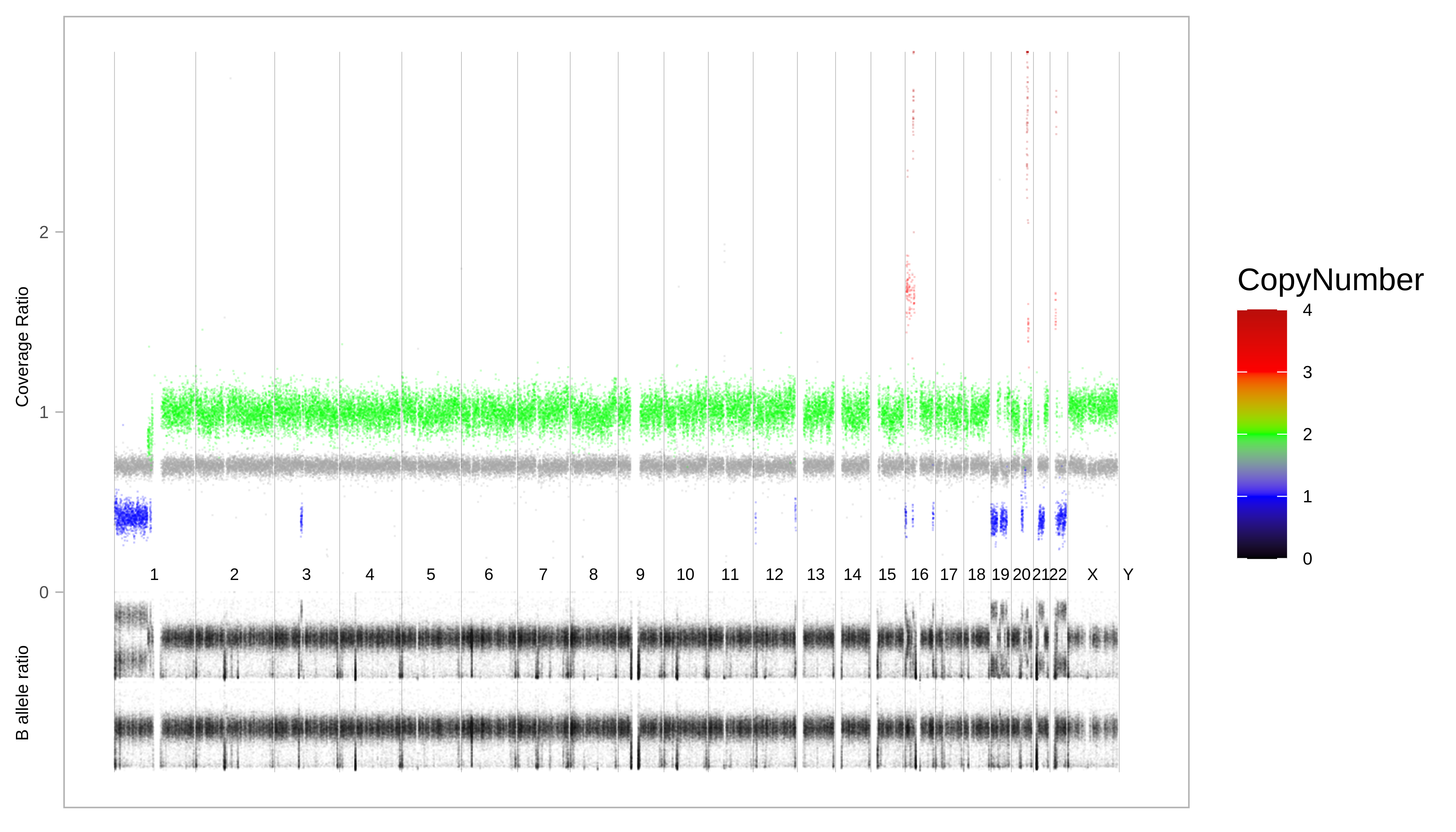


**D:** P2233_106T - A rhabdoid meningioma with highly rearranged genome profile


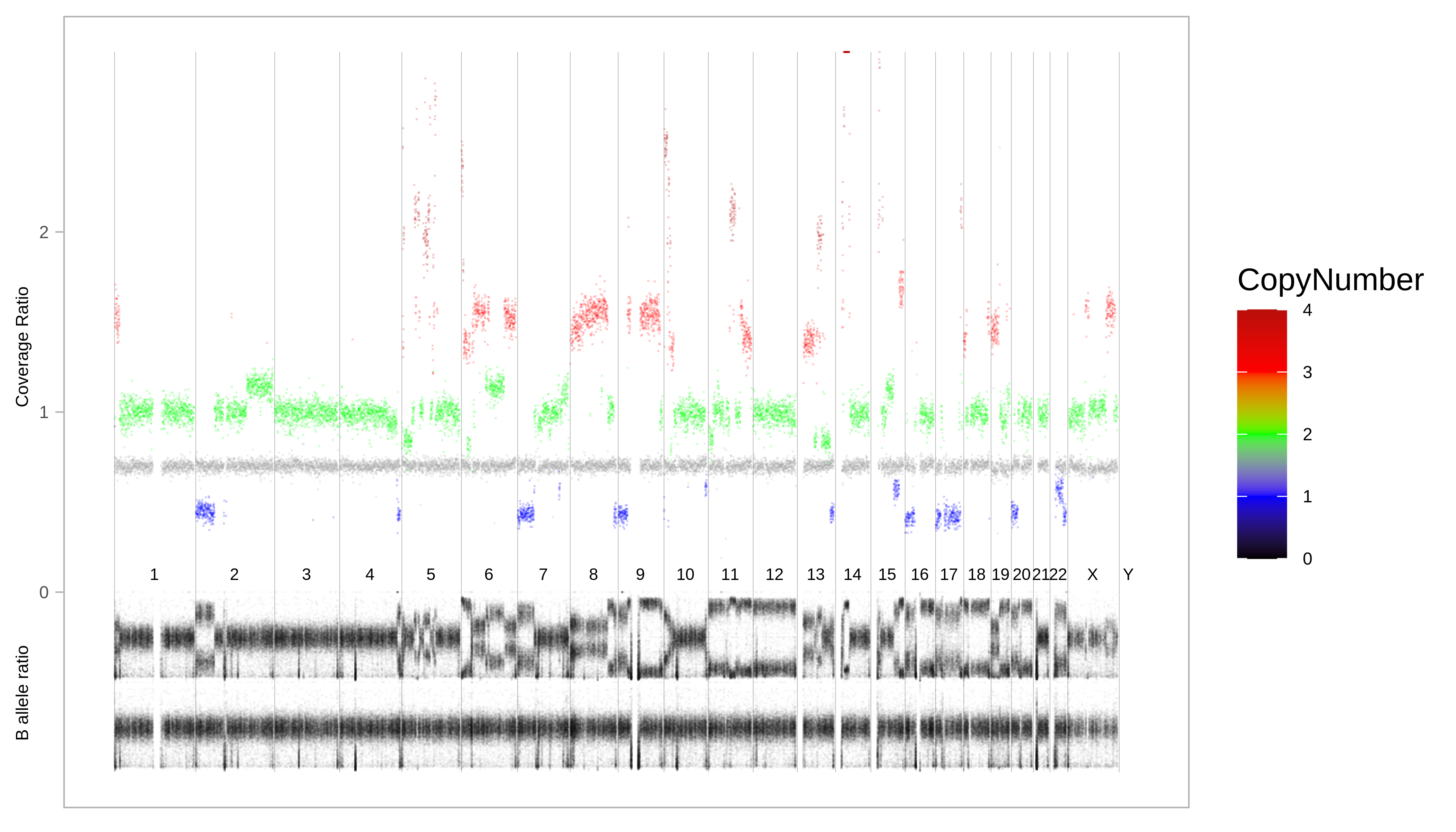

Supplement: Supplementary file 7 — Additional file 7: Figure S3. Whole genome copy number profiles for selected samples with multiple structural variants. The tumor copy number status is indicated in different colors according to a scale (0 to 4) shown to the right in the figure. The copy number profile for the paired normal sample is shown in gray below the tumor track. A Highly rearranged genome profile in a tumor diagnosed as PNET (P2233_101T) and classified by methylation profiling as GBM IDH wild-type (though with lower score, 0.61). TP53 inactivation due to a somatic splice donor mutation plus LOH was observed. Numerous CNVs affecting almost all chromosomes and several focal amplifications were also detected, including amplification of EGFR on 7p11.2, KRAS on 12p12.1 (~70 DNA copies) and a nearby duplication (> 50 DNA copies) creating a PLEKHA5::PIK3C2G fusion (Table 1, Additional file 2: Table S1). B A GBM (P2233_118T) that could not be assigned to a specific class by the methylation classifier showed many SVs, including numerous focal deletions within chrX, focal duplications and deletions on chr15, as well as a focal RB1 homozygous deletion (an extremely rare event in pediatric HGG). SV involving NTRK3 and a predicted fusion involving RERE::SLC2A5 were detected (Table 1, Additional file 2: Table S1). C An atypical meningioma (P2233_116T) displaying several copy number aberrations, including loss of chr22, which is a frequent event in pediatric meningiomas [32]. Multiple interchromosomal translocations were also observed on chr16, 19, 20 and 22, two of which involved NF2 (chr22:29638300 T]CHR12:131449186] and chr22:29645023 [CHR3:86137311[T) and led to NF2 transcript ablation. The tumor also displayed a focal amplification on 20q13.13, an event associated with progression and metastasis in several cancers. This locus encompasses, among others, ZFAS1, encoding a noncoding RNA that regulates the expression of genes involved in differentiation. An oncogenic role for ZFAS1 lncRNA has been describ [file 12967_2023_4178_MOESM7_ESM.docx]
